# An endangered new species of seasonal killifish of the genus *Austrolebias* (Cyprinodontiformes: Aplocheiloidei) from the Bermejo river basin in the Western Chacoan Region

**DOI:** 10.1371/journal.pone.0196261

**Published:** 2018-05-16

**Authors:** Felipe Alonso, Guillermo Enrique Terán, Pablo Calviño, Ignacio García, Yamila Cardoso, Graciela García

**Affiliations:** 1 Instituto de Bio y Geociencias del NOA (IBIGEO)-CONICET, 9 de julio 14, Rosario de Lerma, Provincia de Salta, República Argentina; 2 Grupo de Investigación y Conservación de Killis (GICK), Calle, Berisso, Buenos Aires, República Argentina; 3 Unidad Ejecutora Lillo (UEL)-CONICET-Fundación Miguel Lillo, Miguel Lillo 251, San Miguel de Tucumán, CEP, Tucumán, Argentina; 4 Instituto de Limnología “Dr. Raúl Ringuelet” (ILPLA)—UNLP–CONICET, La Plata, Buenos Aires, Argentina; 5 Laboratorio de Sistemática y Biología Evolutiva, Facultad de Ciencias Naturales y Museo, UNLP–CONICET, La Plata, Buenos Aires, Argentina; 6 Sección Genética Evolutiva, Facultad de Ciencias, Universidad de la República, Montevideo, Uruguay; Universita degli Studi di Roma La Sapienza, ITALY

## Abstract

*Austrolebias wichi*, new species, is herein described from seasonal ponds of the Bermejo river basin in the Western Chacoan district in northwestern Argentina. This species was found in a single pond, a paleochannel of the Bermejo River, which is seriously disturbed by soybean plantations surrounding it. Despite intensive sampling in the area, this species was only registered in this pond where it was relatively scarce. Therefore, we consider this species as critically endangered. This species is the sister species of *A*. *patriciae* in our phylogenetic analyses and is similar, in a general external aspect, to *A*. *varzeae* and *A*. *carvalhoi*. It can be distinguished among the species of *Austrolebias* by its unique color pattern in males. Additionally, from *A*. *varzeae* by presenting a supraorbital band equal or longer than the infraorbital band (vs. shorter) and from *A*. *patriciae* by the convex dorsal profile of head (vs. concave). Further diagnostic characters and additional comments on its ecology and reproduction are provided.

## Introduction

The genus *Austrolebias* Costa comprises 48 valid species [1, 2, 3] inhabiting seasonal ponds at the Southern portion of Neotropics in La Plata drainage, with one speciesfrom the Amazon basin in the Bolivian Chaco. Species of this genus are known from the lowlands in the Chaco-Pampasic floodplains of Argentina, Bolivia, and Paraguay. The genus also inhabits Uruguay (Uruguay river drainage and costal drainages) and Southern Brazil (Uruguay and Iguazú river drainages and costal drainages), where it presents its highest species diversity, probably due to the complex geological history of these regions [4, 5, 6].

Six species of *Austrolebias* have been recorded from the Chaco Freshwater ecoregion *sensu* Hales and Petry [7]. *Austrolebias accorsii* Nielsen & Pillet, 2015 may also be listed here, because although it inhabits the Amazon basin, the area in which this species occurs has been in contact with La Plata basin and only recently, in a geological timescale, was captured by the Amazon system [1]. *Austrolebias* species in the Chacoan region can be geographically divided in two non-overlapping groups, with two species from the Western Chacoan (WC) region (*sensu* Morrone [8]): *A*. *monstrosus* (Huber 1995) and *A*. *vandenbergi* (Huber 1995) [9, 10, 11]; and four species from the Eastern Chacoan (EC) region, of which two are endemic: *A*. *toba* Calviño 2006 and *A*. *patriciae* (Huber 1995), and two species shared with the pampasic region: *A*. *bellottii* (Steindachner 1981) and *A*. *nigripinnis* (Regan 1912).

In the present work we describe a third *Austrolebias* species from the Western Chacoan region which is closely related to *A*. *patriciae*, present in the Eastern Chacoan region. Colour pattern and general morphology resembles also *Austrolebias varzeae* Costa, Reis & Behr, 2004, from the upper Uruguay basin and *A*. *carvalhoi* (Myers, 1947), from the upper Iguazú basin. Also we analyzed the phylogenetic relationship of this new taxon based on morphology and a molecular marker.

## Material and methods

Appropriate actions were taken to minimize pain or discomfort of fish, and this study was conducted in accordance with international standards on animal welfare, as well as being compliant with national regulations and the “Comité Nacional de Ética en la Ciencia y la Tecnología” of Argentina. Specimens were euthanized by immersion in an anesthetic solution (0.1% 2-phenoxyethanol), and then fixed in a 4% formaldehyde for one week, washed in water for one day and transferred to a 70% ethanol solution for preservation. These procedures are approved by the ethical use of animals of Instituto de Bio y Geociencias del NOA (IBIGEO) that consider animal welfare regulations. Collection permit was granted by Secretary of Environment and Sustainable Development (Disp 0335). Descriptions of color patterns are based on photographs of both sides of live individuals. Measurements and counts follow Costa [12]. Measurements are presented as percentages of standard length (SL), except for those related to head morphology, which are expressed as percentages of head length. Fin-ray counts include all elements. Number of vertebrae and gill-rakers were recorded only from the cleared and stained specimen; the compound caudal centrum was counted as a single element. The osteological preparation was made according to Taylor & Van Dyke [13]. Terminology for cephalic neuromast series follows Costa [14]. The abbreviation c&s means specimens cleared and stained for bone and cartilage.

Type material is deposited in the ichthyological collections of Fundación Miguel Lillo (CI-FML), San Miguel de Tucumán, and Instituto de Bio y Geociencias del NOA (IBIGEO-I), Rosario de Lerma, both from Argentina. Comparative material is provided as supplementary file.

Three individuals of *Austrolebias wichi* n.sp. were sampled and stored separately in 95% ethanol. DNA was extracted using peqGOLD Tissue DNA Mini Kit (PeqLab). A 793-bp fragment of Cyt-b was amplified using primers CB3-H and Gludg-L [15] and PCR cycle profile: 93°C for 1 min, 45°C for 1 min, 72° for 1 min; 30 cycles following Garcia *et al*. [16]. Bidirectional sequencing was performed by the company MAGROGEN (Korea). New sequences were deposited in GenBank under accession numbers: MH058098, MH058099, MG811839-40 and MG811840. We also obtained published data for *A*. *patriciae* (Genbank acc. number: FJ826897) from the type locality. Sequences were edited and aligned using BioEdit 7.0.1 [17]. Genetic distance among sequences was estimated using Kimura 2-P model in Mega.7 [18].

## Phylogenetic analysis

The new taxon was added to the morphological matrix of Costa [4] (see S2 Appendix) and its phylogenetic relationships evaluated through a parsimony analysis. The analysis was done under implied weighting [19, 20] with K = 6. Support measures were calculated through symmetric resampling and expressed as GC values [21]. Also, a phylogenetic analysis was made combining the data from Costa [4] with the available information on cytochrome b (cytb), 36 species of *Austrolebias* were retrieved from GenBank and the following outgroup taxa: *Nematolebias whitei*, *Neofundulus paraguayensis* (see S3 Appendix). The consensus of the sequences for species when more than one was available was used. These consensuses were performed with the aim of considering molecular polymorphisms as is regularly done with morphological characters. A multiple sequence alignment was performed by using the ClustalW tool implemented in MEGA7 [18]. All searches have been performed with TNT [22]. Some multistate character of the dataset of Costa [4] are interpreted herein as additive characters: 4, 19, 21, 23, 26, 29, 33, 42, 64 72. Additionally, following the methodology of García *et al*. [23] we performed a Bayesian Inference (BI) phylogenetic analysis of the cyt b sequences alone that is presented as supporting information in S4 Appendix.

### Nomenclatural acts

The electronic edition of this work follows the requirements of the International Code of Zoological Nomenclature, and hence the new names contained herein are available under that Code from the electronic edition of this article. This published work and the nomenclatural acts it contains have been registered in ZooBank, the online registration system for the ICZN. The ZooBank LSIDs (Life Science Identifiers) can be resolved and the associated information viewed through any standard web browser by appending the LSID to the prefix “http://zoobank.org/”. The LSID for this publication is: urn:lsid:zoobank.org:pub:3CF334BA-3A3C-494F-BC1E-E9320EB82152.

### *Austrolebias wichi*, new species

**Fig 1:**
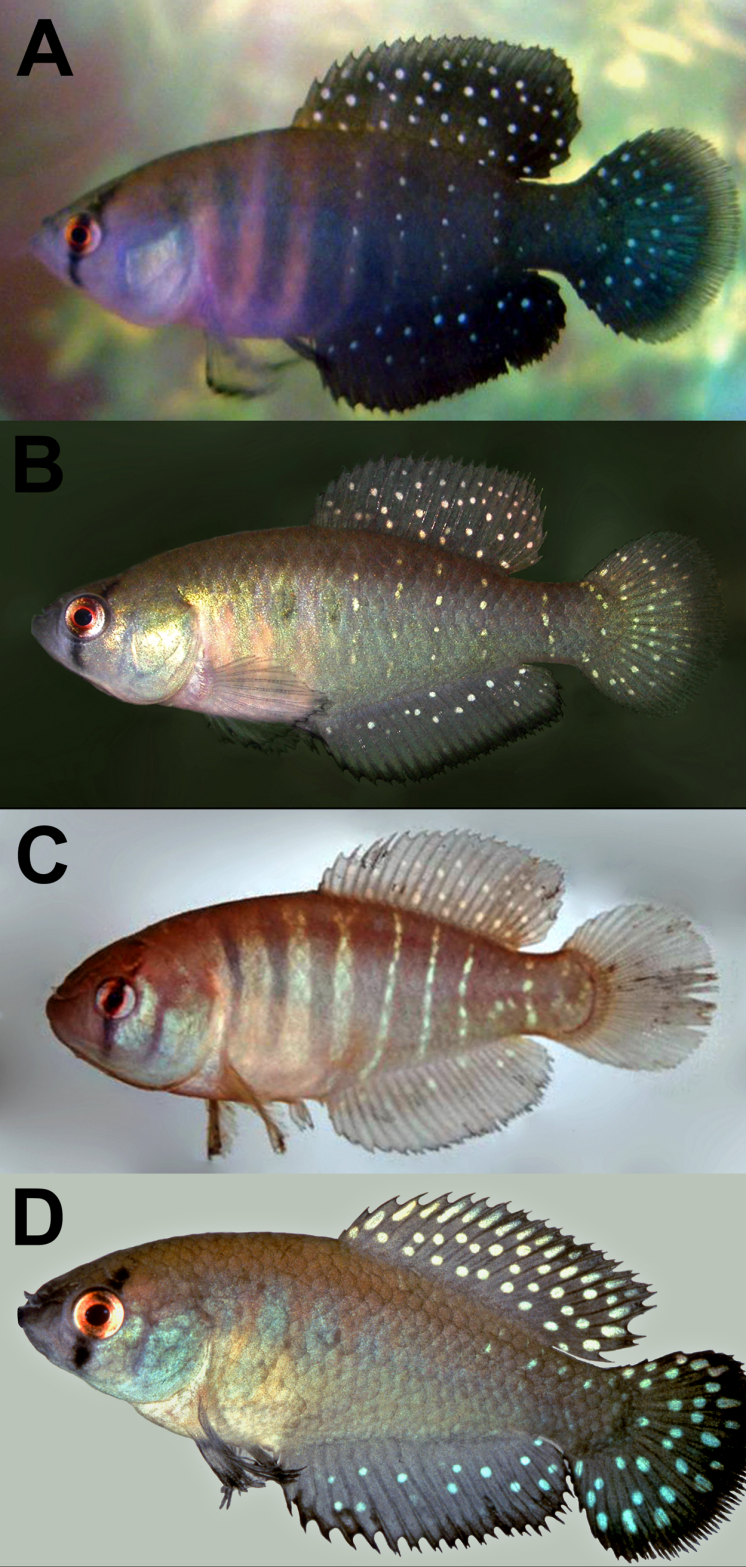
Live pictures of males in left lateral view. (A-B) *Austrolebias wichi* sp. nov. (C) *Austrolebias varzeae*, picture by Matheus Volcan; D) *Austrolebias patriciae* from type locality, not preserved, picture by Daniel W. Fromm.

**Fig 2:**
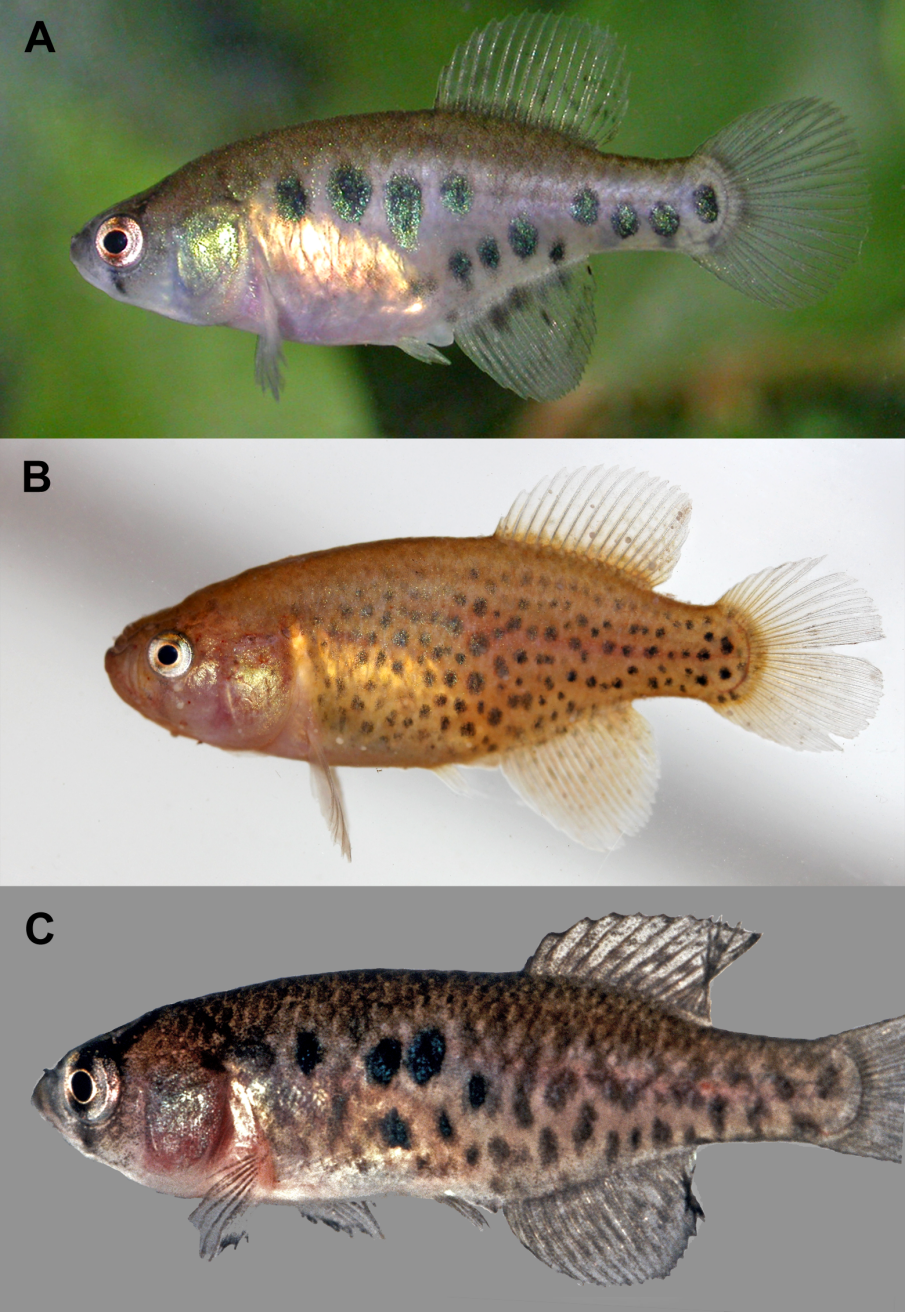
Live pictures of females in left lateral view. (A) *Austrolebias wichi* sp. nov. (B) *Austrolebias varzeae*, picture by Matheus Volcan; (C) *Austrolebias patriciae* from type locality, not preserved, picture by Daniel W. Fromm.

**Fig 3:**
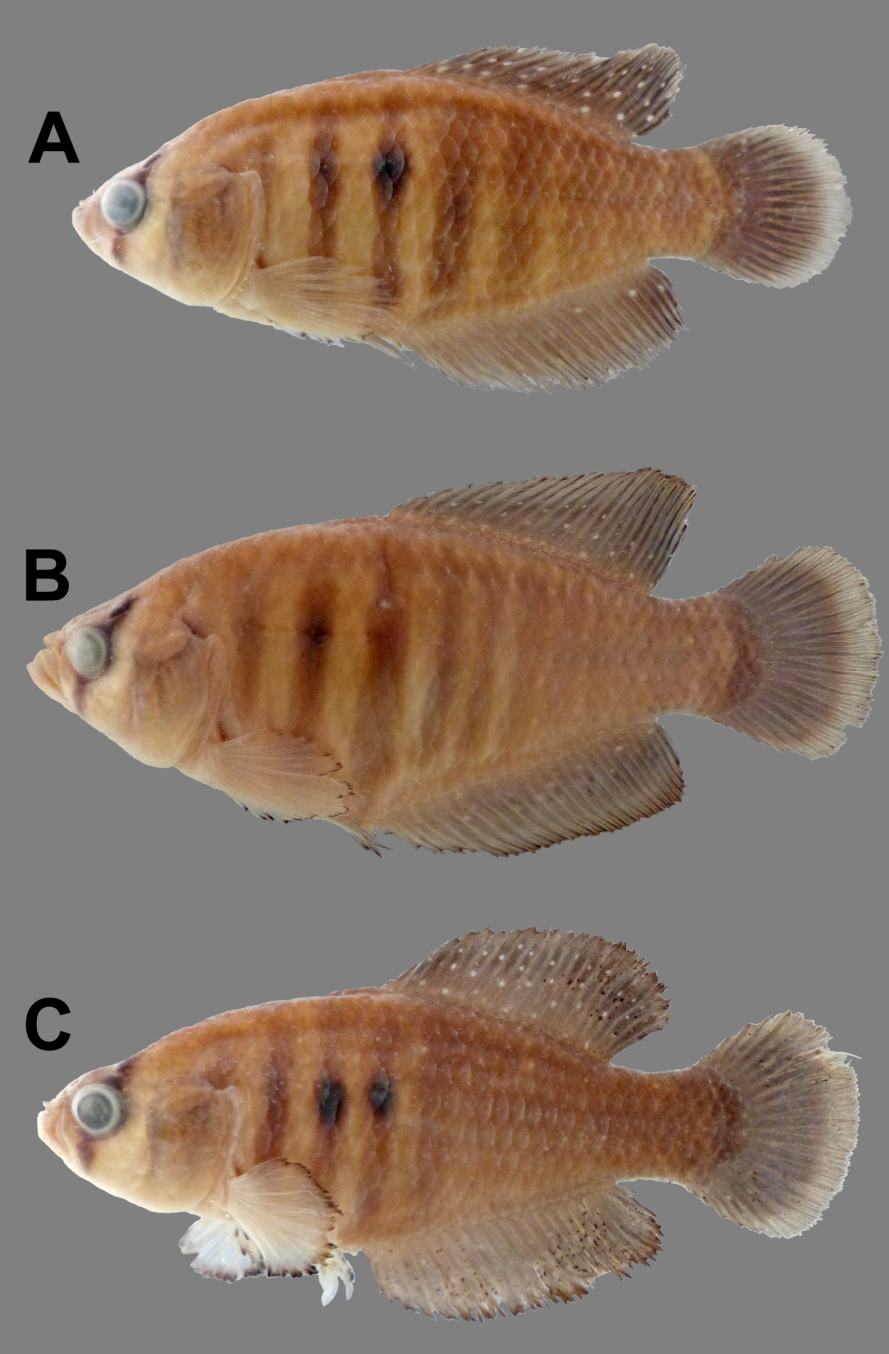
Male specimens preserved. (A) Holotype, 40.7mm. IBIGEO-I 443; (B) paratype, 51.6mm SL, IBIGEO-I 444 and (C) paratype, 41.4mm SL, IBIGEO-I 444.

**Fig 4:**
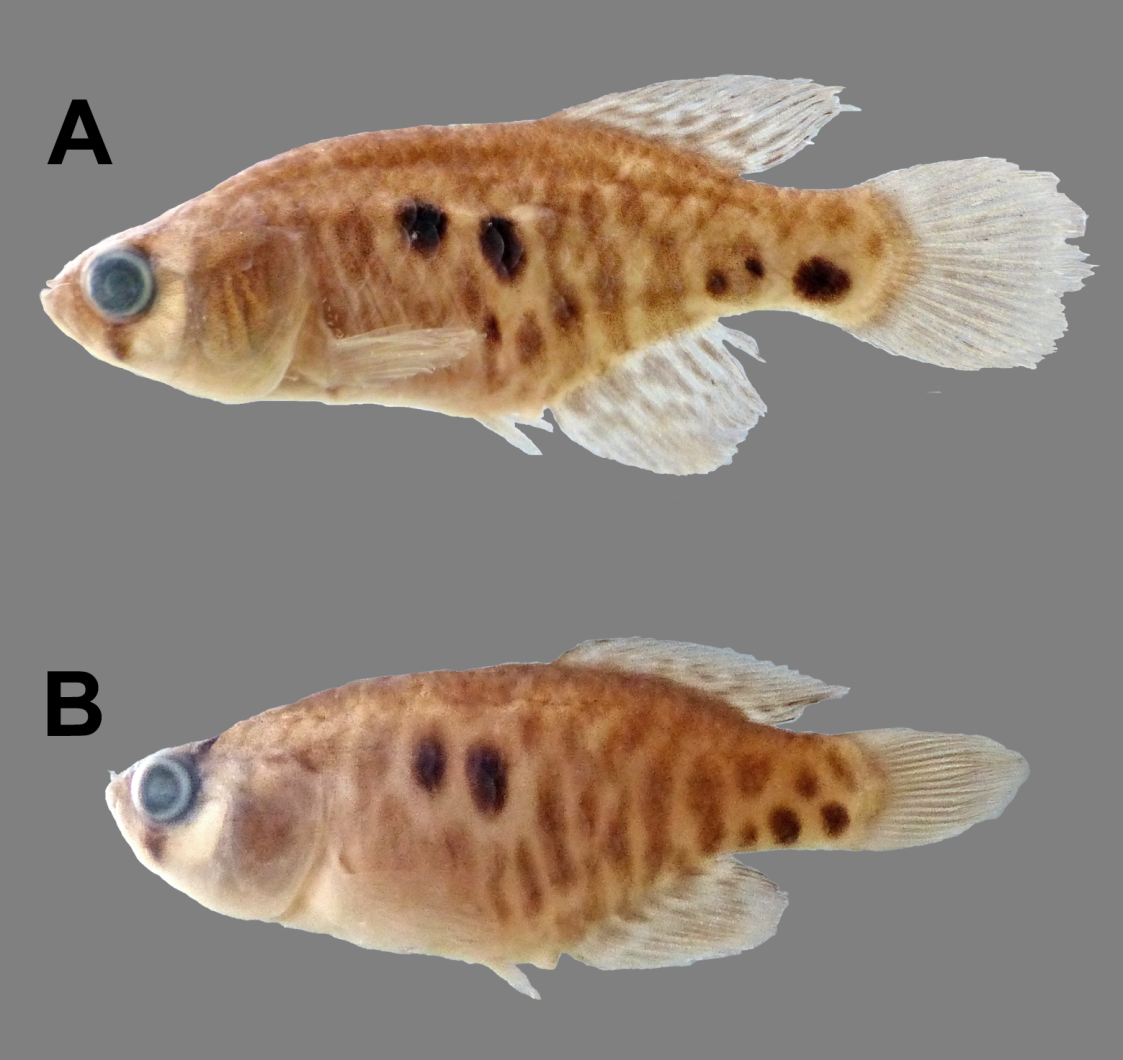
Female specimens preserved, IBIGEO-I 444, paratypes. (A) 30.1mm SL; (B) SL 25.6mm SL.

urn:lsid:zoobank.org:act:5D3E79D4-1DDA-4D29-8A7D-BF087801AE99

(Figs 1, 2, 3A, 3B and 4A), Tables 1 and 2.

**Table 1:** Morphometric data for *Austrolebias wichi* n. sp.: Holotype and paratypes (n = 18). SD: standard deviation. Range, Mean and SD include holotype.

	Males (n = 11)				Females (n = 7)		
	Holotype	Range	Mean	SD	Range	Mean	SD
Standard length	40.1	24.7–51.6	35.8	8.5	25.5–33.3	28.7	2.7
Percents of standard length							
Body depth	40.3	34.0–42.4	37.5	2.7	32.6–38.1	35.2	1.9
Caudal peduncle depth	16.8	14.3–16.8	15.7	0.7	12.7–16.2	14.5	1.0
Pre-dorsal length	53.3	50.7–53.4	52.4	0.9	58.1–64.6	61.9	2.6
Pre-pelvic length	45.1	42.1–47.5	45.5	1.6	47.1–53.7	51.6	2.3
Length of dorsal-fin base	36.4	34.5–42.5	37.6	2.6	25.2–31.4	26.7	2.2
Length of anal-fin base	40.9	35.5–43.5	39.6	2.3	21.2–27.4	24.1	2.1
Caudal-fin length	24.5	20.7–27.8	24.3	2.7	23.8–27.7	25.5	1.4
Pectoral-fin length	22.0	16.9–25.5	21.1	2.6	21.3–25.3	23.1	1.4
Pelvic-fin length	10.7	8.3–11.9	10.0	1.3	9.1–10.8	9.8	0.6
Head length	33.0	29.2–33.6	31.4	1.5	31.6–35.1	33.3	1.1
Percents of head length							
Head depth	77.1	69.8–89.4	81.2	5.4	70.7–79.3	73.3	2.9
Head width	61.8	61.8–71.4	67.9	2.9	61.7–68.9	65.3	2.9
Snout length	25.6	22.9–33.3	27.7	3.1	20.4–28.6	24.0	2.8
Lower jaw length	37.9	33.2–41.3	37.8	2.2	30.9–34.1	32.6	1.3
Eye diameter	24.2	24.9–28.3	26.4	1.2	24.6–29.2	26.7	1.7

**Table 2:** Mean historical monthly precipitations and air temperatures at Hickmann (23km east from type locality). Data obtained from Arias & Bianchi [26].

	Jan	Feb	Mar	Apr	May	Jun	Jul	Aug	Sep	Oct	Nov	Dic	Annual
Precipitation / mm	123	99	80	38	7	4	3	1	8	40	71	104	**579**
Mean Temperature / °C	26.8	25.7	24.3	21.3	18.6	15.5	15.4	17.3	20.2	23.8	25.3	26.5	**21.7**

#### Holotype

IBIGEO-I 443, 40.1mm SL, male; Argentina: Salta Province: Departamento de San Martín: near Padre Lozano town: temporary pond at the side of RP 53, Bermejo river basin, Paraná river basin, 23°13'2.81"S 63°49'12.66"W, altitude 274 m above sea level; F. Alonso and P. Calviño, January 2007.

#### Paratypes

IBIGEO-I 444, 2 males (41.4, 51.6mm SL), 2 females (25.6, 30.1mm SL). CI-FML 7285, 3 males (30.7–45.4mm SL), 2 females (30.2, 33.3mm SL). Collected with the holotype.

IBIGEO-I 445, 3 males (24.7–32.3mm SL), 3 females (26.2–28.7). CI-FML 7284 1 male 30.4mm SL. Same locality and collectors as the holotype. January 2010.

#### Diagnosis

Distinguished from all other congeners except from *Austrolebias patriciae* by a supraorbital bar longer or equal than infraorbital bar (vs. always shorter than infraorbital bar). *Austrolebias wichi* can be distinguished from *Austrolebias patriciae* by head dorsal profile on lateral view concave (vs. convex), the absence of filamentous rays markedly overpassing the interradial membrane distal margin of dorsal and anal fin in adult males (*vs*. present), by presenting small numerous whitish dots on unpaired fins in males (vs. fewer and bigger), infraorbital and supraorbital bands thinner than pupil and pointed distal portion (vs. equal or wider than pupil and rounded distal portion), dorsal-fin origin posterior to anal fin origin in females (vs. anterior) (Fig 1).

Female colour pattern similar to *A*. *patriciae*, with grey pinkish background having irregular grey blotches and some dark blue blotches over the caudal peduncle and body flank and differing from *A*. *varzeae*, which presents an orange background with minute black and grey relatively rounded, irregular blotches (Fig 2), and from *A*. *araucarianus* which presents a yellowish brown pale flank, with vertically elongated dark grey to black spots, often forming short narrow bars [24].

#### Description

Morphometric data is shown in Table 1. Males larger than females, largest examined male 51.6 mm SL, largest female 33.3 mm SL. Dorsal profile on lateral view concave from snout to vertical through anterior margin of operculum and concave from this point to posterior end of dorsal-fin base, approximately straight on caudal peduncle. Ventral profile on lateral view convex from lower jaw to end of anal-fin base, nearly straight on caudal peduncle. Greatest body depth at level of pelvic-fin base. Body moderately deep and laterally compressed. Snout blunt and jaws short. Tip of both dorsal and anal fins rounded. Anteromedian rays of anal fin of females not lengthened. Caudal fin rounded. Pectoral fins rounded, posterior margin on vertical through base of 2^nd^ anal-fin ray in males, through pelvic-fin base in females. Tip of each pelvic fin reach base of 2^nd^ anal-fin ray in males, reach urogenital papilla in females. Pelvic-fin bases united. Urogenital papilla not attached to anal fin. Dorsal-fin origin usually slightly posterior to anal-fin origin in males, on vertical between base of 1^st^ and 2^nd^ anal-fin rays, or sometimes slightly anterior; dorsal-fin origin anterior to anal fin origin in females; dorsal-fin origin between neural spines of 7^th^ and 9^th^ vertebrae in males, between neural spines of 10^th^ and 11th vertebrae in females. Anal-fin origin between pleural ribs of 7^th^ and 8^th^ vertebrae in males, between pleural ribs of 10^th^ and 11^th^ vertebrae in females. Six branchiostegal rays. Dermosphenotic ossification absent. Urohyal deep. Total number of vertebrae 27–29, 11–12 precaudal. Gill rakers in first branchial arch 3+11. Basihyal subtriangular, width about 50% of its length; basihyal cartilage about 50–60% of total basihyal length. One to three teeth on second pharyngobranchial.

Dorsal-fin rays 20–24 in males, 16–19 in females; anal-fin rays 23–27 in males, 19–21 in females; caudal-fin rays 21–25; pectoral-fin rays 10–12; pelvic fin rays 5 (one female with 4).

#### Scales large and cycloid

Trunk and head fully scaled, except ventral surface of head. No scales on dorsal and anal-fin bases; two rows of scales on caudal-fin base. Frontal squamation H; E-scales overlapping medially; scales arranged in transverse pattern. Longitudinal series of scales 25–29, scales regularly arranged; transverse series of scales 11–14; scale rows around caudal peduncle 13–16. One contact organ, sometimes 2, on each scale on mid and dorsal region, 3–5 in ventral region and none on opercles in males. No contact organs on fins.

#### Coloration in life

Male (Fig 1A and 1B). Green to dark green bluish in unpaired fins and body coloration forming vertical bars in body, more intensively colored on caudal peduncle and caudal-fin proximal portion. Three wide vertical bars anterior to anal fin, separated by a space similar to its width of a pale pink hue. Neither of these bars reaches body dorsum. The posterior most of these bands wider than the rest, approximately as wide as eye diameter. Two humeral blotches over the two posterior bands in some individuals and more conspicuous in juveniles. Most posterior blotch more conspicuous. First anterior vertical bar on body not conspicuous, sometimes not visible, from operculum posterior margin to dorsal region of body, slightly curved anteriorly in dorsal region. Four vertical lines of iridescent dots between dorsal and anal fin, sometimes over a light pink background that separates vertical bands, sometimes vertical bands not discernible in this region. One vertical line of iridescent dots over caudal peduncle. Dorsal and caudal fin with numerous small iridescent whitish dots, only on basal and medial portion of anal fin. Caudal fin with a grey distal band without dots. Anal, pectoral and pelvic fins with black distal margin. Black anal-fin distal margin of about 15–25% of rays length.

Females (Fig 2): Overall color light pinkish with violet iridescence, with a variable number of dark gray or dark brown irregular blotches, usually presenting one to five more conspicuous dark blue and larger irregular blotches arranged on the anterocentral portion of the flanks and some over the anal fin and caudal peduncle. Belly pale golden. Opercular region pale greenish golden. Unpaired fins hyaline with dark grey faint irregular blotches on the anal and dorsal fins. Caudal, pectoral, and pelvic fins hyaline. Iris white, with a dark vertical bar through the center of eye surrounded by red at it sides. Supraorbital and suborbital bars dark grey.

#### Colouration in alcohol

Overall coloration of specimens in alcohol similar to alive individuals but beige background with grey brown bands and unpaired fins with hyaline dots in males and beige background with scattered brown blotches over body and hyaline fins in females (Figs 3 and 4).

#### Distribution and conservation status

**Fig 5:**
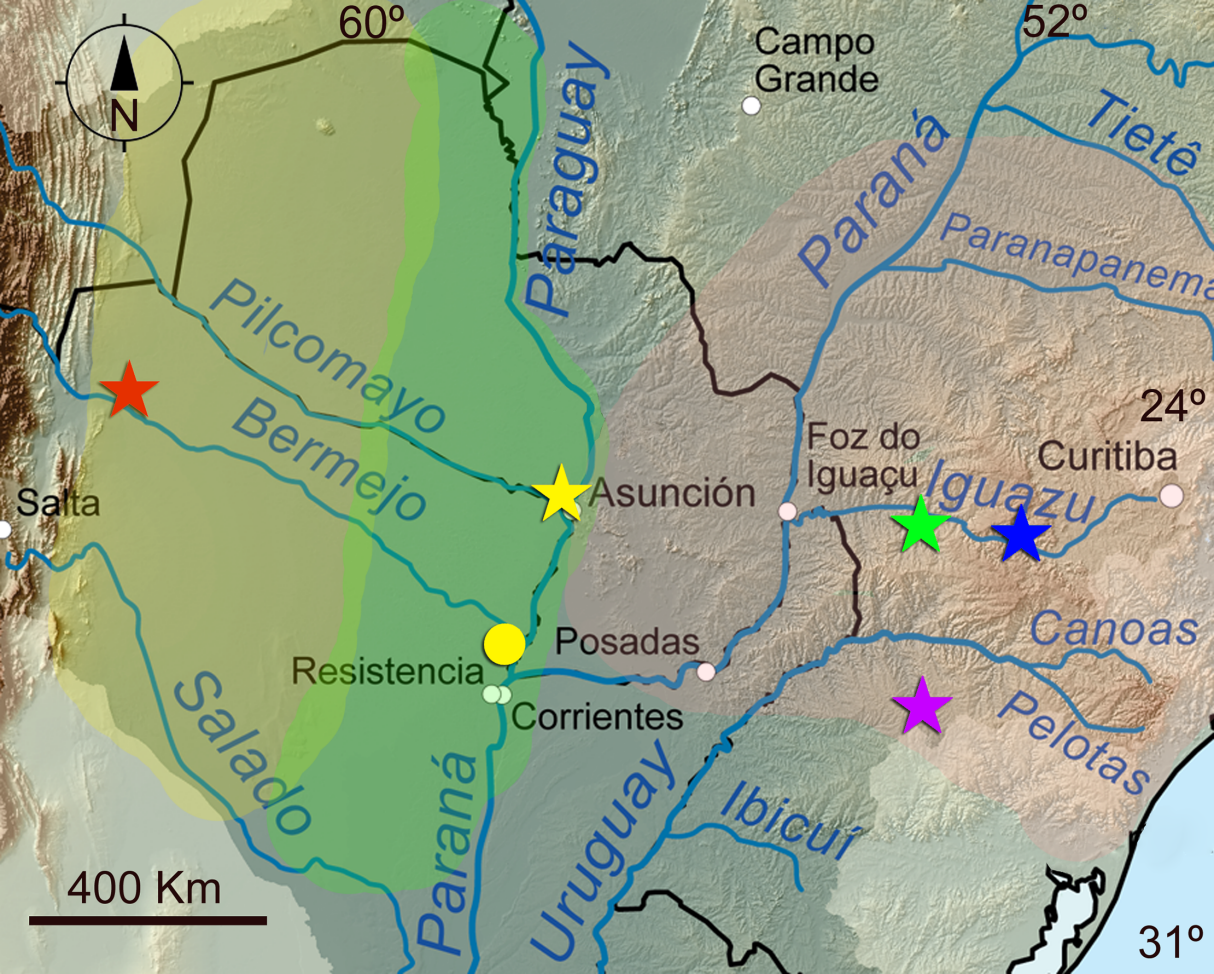
Map showing the known distribution of *Austrolebias* species treated in this work. Stars represent type localities and circles represent other records. Each symbol may represent more than one record. The following colors of symbols correspond to: red, *Austrolebias wichi* sp. nov.; yellow, *A*. *patriciae*; fuchsia, *A*. *varzeae*; blue, *A*. *carvalhoi*; and green, *A*. *araucarianus*. Yellow area approximately represents Western Chacoan (WC) district, green area represents Eastern Chacoan (EC) district, and pink area represents Parana region.

*Austrolebias wichi* is only known from its type locality, a seasonal paleochannel from the Bermejo River basin (Paraná River basin) in Northwestern Argentina, near Padre Lozano town, aside RP 53 (23°13'2.81"S, 63°49'12.66"W, altitude 274 m above sea level) (Fig 5). Although this area has been object of fish collecting efforts at least since the 1950’s with many lots collected by S. Pierotti deposited in the main ichthyological collections of Argentina, it has not been recorded elsewhere. We have explored most of the area in the WC region North and South of the Bermejo River basin in more than 12 expeditions since 2005 and were not able to collect this species in a different location than its type locality. In some years we were not even able to collect it there and it was always much less abundant than the other syntopic killifishes. The type locality is surrounded by mostly transgenic soybean plantations that regularly use herbicides and other chemicals which most likely impact this environment. In our last visit here on March 2017 we were not able to collect any specimen of this species. Following the Categories & Criteria of the IUCN [25], we consider this species as critically endangered due to the small area of distribution and the intense recent decline of the population meeting Criteria V. B. a. b. c.

#### Etymology

The name *wichi* is a reference to the occurrence of the new species in the Western Chacoan region where the Wichí indigenous people inhabits in several settlements very close to the type locality.

#### Ecology

**Fig 6:**
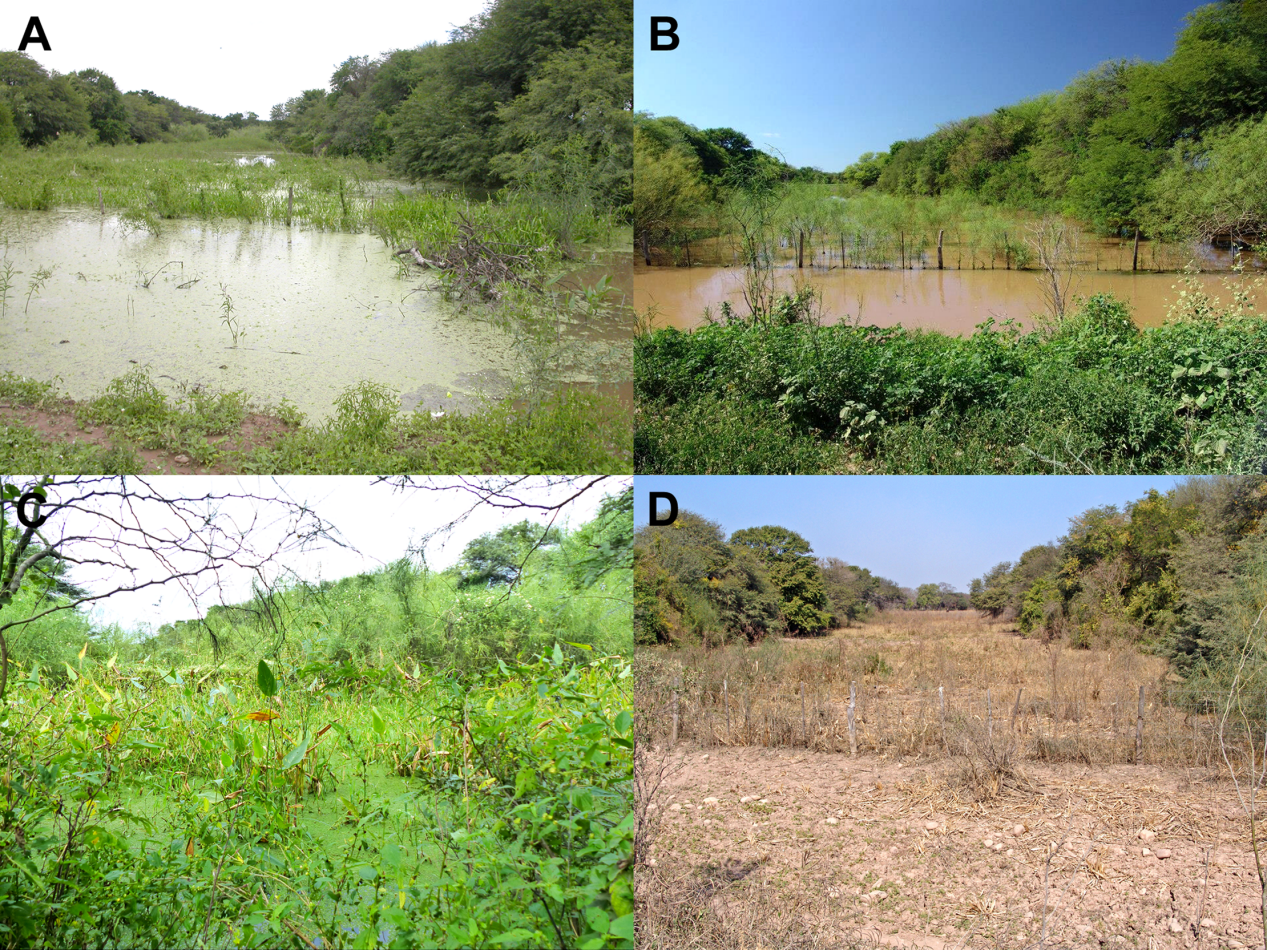
Type locality of *Austrolebias wichi* sp. nov. (A) January 2006. (B) January 2014. (C) April 2017. (D) August 2012.

The ponds in the region have marked dry and wet seasons; the rains are concentrated during the summer, with about 75% of the total rains concentrated from December to March, and almost no rains from May to September [26] (Table 2). This determines that the seasonal ponds present water approximately from December to April, depending on the pond and the variability among years, (pers. obs.) (Fig 6).

The seasonal aquatic environment where the new species was collected is part of a long paleochannel which is interrupted by a road. Despite intensive sampling efforts in this area and in the Western Chacoan region we were only able to collect this species in the portion of the paleochannel immediately next to the road. Physicochemical parameters measured on January 2006 where pH 6,9 and a conductivity of 70 μsiemens/cm. This environment generally presents abundant aquatic vegetation. Other syntopic killifish species are: *Papiliolebias bitteri* (Costa 1989) and *Trigonectes aplocheiloides* Huber, 1995, which are the most abundant species, followed in abundance by *Austrolebias vandenbergi* (Huber, 1995) and *A*. *wichi*, which is very scarce in this environment, and some years we could not even collect a single specimen of this species while there were other annual fishes in the pond. Also, very few *Neofundulus paraguayensis* (Eigenmann & Kennedy, 1903) were collected in this pond. Nearby, a couple of hundred of metres from this environment there is another pond where we collected *Austrolebias monstrosus* (Huber, 1995) but this species was not found syntopically with *A*. *wichi*. There are many seasonal ponds in this area where annual fish are very abundant; nevertheless, the only place where we found *A*. *wichi* is the type locality. The only noticeable difference between this environment and other seasonal ponds in the area may be that this is a very profound (about 1 meter depth) and big environment.

**Fig 7:**
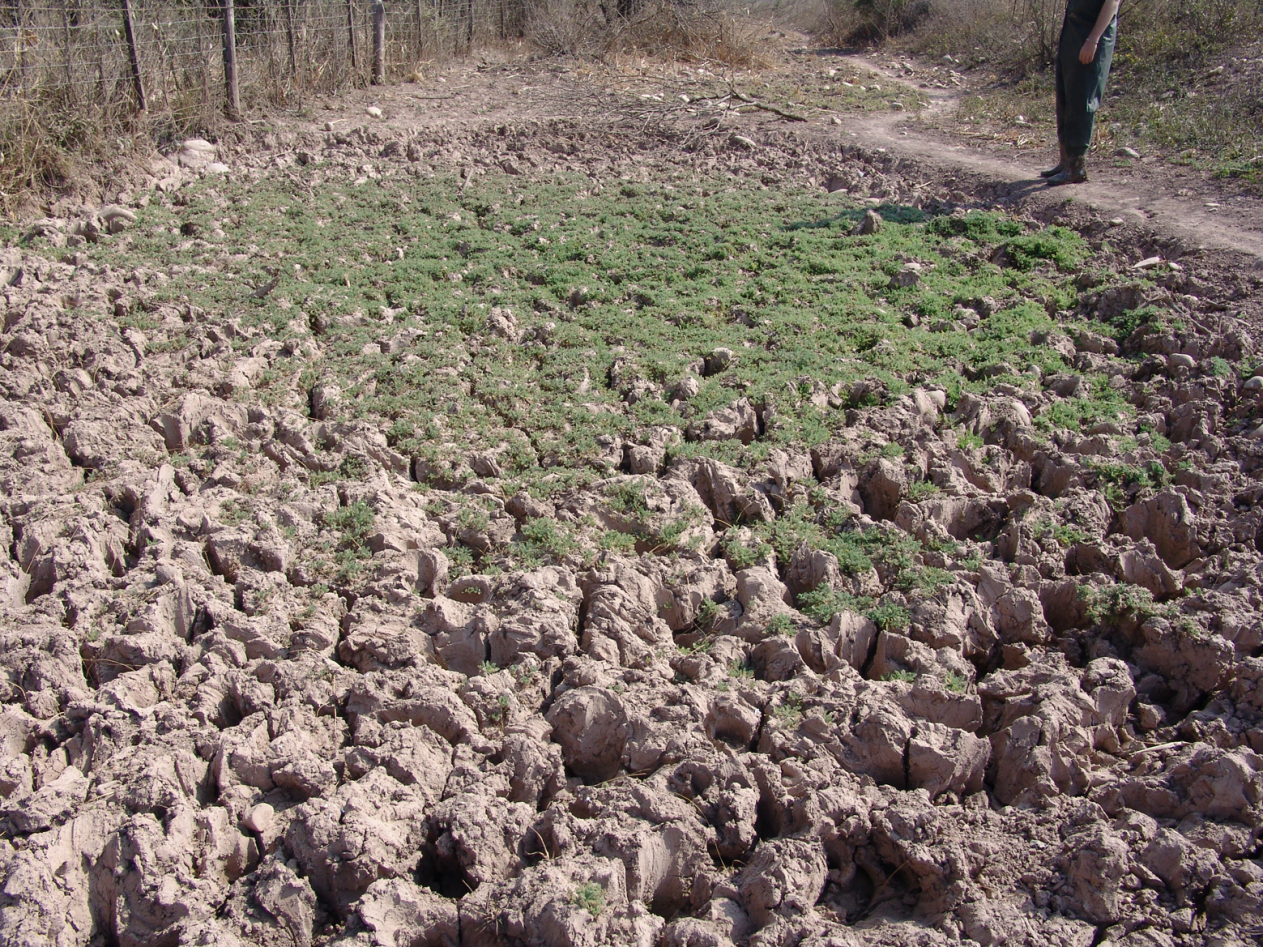
Detail of the bottom of the pond where *Austrolebias wichi* n. sp. is found. August 2012. Picture courtesy of Marcos Mirande.

From mid autumn, winter, and spring the pond is completely dry and the top layer of substrate, which consist of slime with some vegetal rests, is very dry (Fig 7). The presence of domestic cattle in this area is evident in the bottom of the dry pond and the impact of this alteration in the bottom structure over the killifish populations is unknown.

#### Behaviour

The reproductive behavior of this species under aquarium conditions is similar to that described for other *Austrolebias* species [27, 28].

#### Egg development

**Fig 8:**
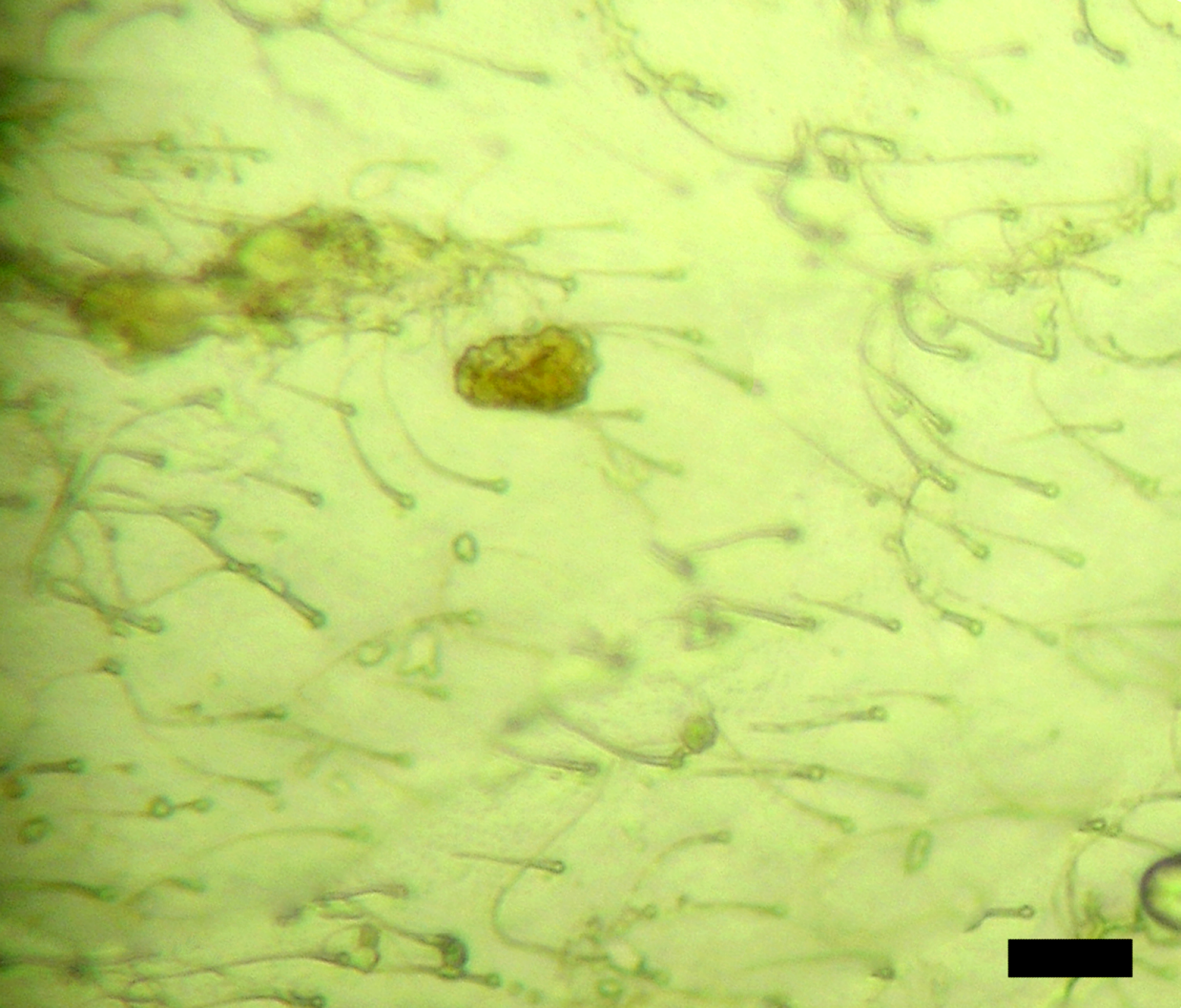
Detail of egg surface of *Austrolebias wichi* n. sp. Bar = 10μm.

As other killifish from the Western Chacoan region (i.e.: *A*. *vandenbergi*, *A*. *monstrosus*, *P*. *bitteri*, etc.) incubation period of the eggs at room temperature before hatching is relatively long, around 6 to 8 months. Character states proposed by Thompson *et al*., [29] are coded for this species herein (Table 3, Fig 8).

**Table 3:** Characters proposed by Thompson *et al*., [29] for the zona pellucida coded for *Austrolebias wichi* n. sp.

**Character number**	**1**	**2**	**3**	**4**	**5**	**6**	**7**	**8**	**9**	**10**	**11**	**12**	**13**	**14**	**15**	**16**
Character state	1	1	0	1	1	0	0	0	0	0	0	0	0	0	0	0

#### Genetic distances

We estimated the genetic distances between *A*. *wichi* and *A patriciae* in order to use this measure as an indirect evidence to sustain these populations as different species. Intraespecific distance in *A*. *wichi* was 0.6% while the genetic distance between this species and *A*. *patriciae* was 2.2%.

#### Phylogenetic analysis

**Fig 9:**
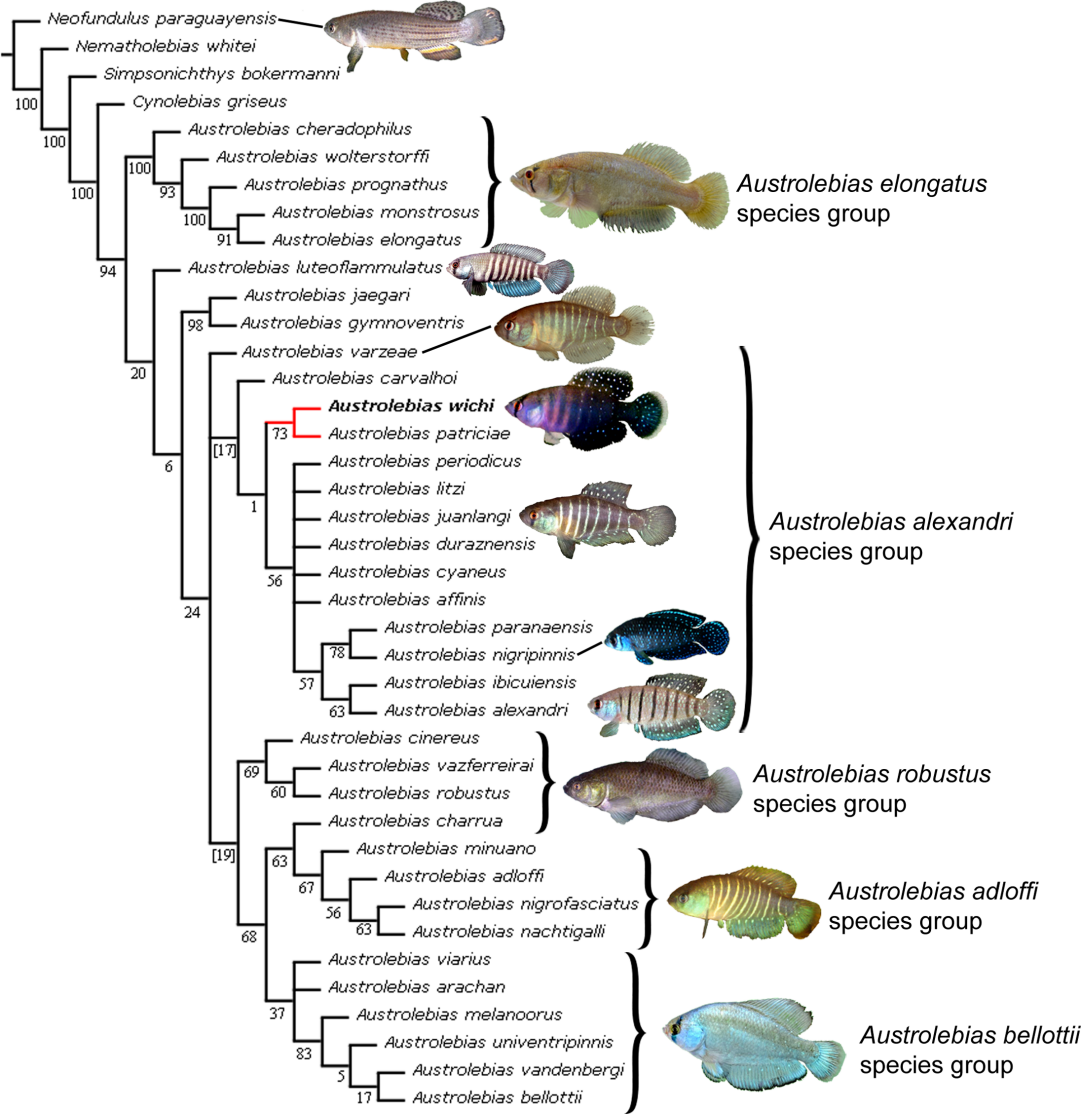
Phylogenetic relationships of *Austrolebias wichi* based on morphological characters. Analysis under implied weighting (K = 6). Numbers above branches denote GC values; negative values are shown in brakets.

**Fig 10:**
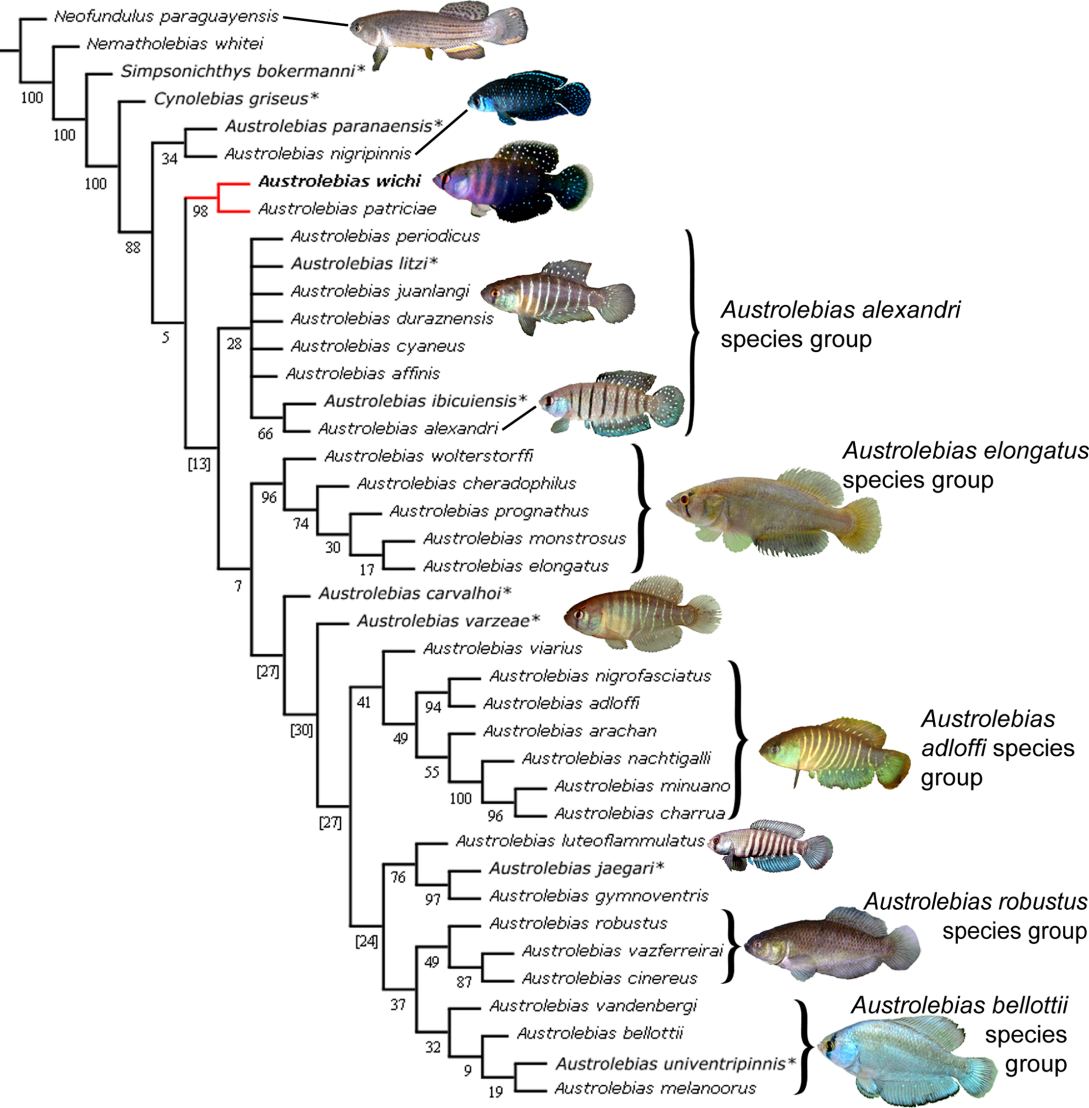
Phylogenetic relationships of *Austrolebias wichi* based on cyt b and morphological characters. Analysis under implied weighting (K = 6). Numbers above branches denote GC values; negative values are shown between brackets. Species with only morphological data available present an asterisk.

The analyses based on morphological data alone (Fig 9) or on morphology and *cytb* (Fig 10) support the close relationship of *A*. *wichi* with *A*. *patriciae*, but the phylogenetic relationships of these two species within the genus are not well supported being the sister group of the *A*. *alexandri* species group, or the sister group of all other *Austrolebias* species, except *A*. *nigripinnis*-*A*. *paranaensis*, in the respective analyses. Nevertheless, those phylogenetic relationships have low support and additional data would be necessary to reach some reliable hypothesis.

## Discussion

The new species posses the diagnostic characters of the genus *Austrolebias* (*sensu* Costa [4]). Additionally, in order to evaluate the phylogenetic position of *A*. *wichi*, we performed a phylogenetic analyses using previous available data ([4]; Genbank) and own data (this study). This is not intended to be an exhaustive phylogeny on the genus but rather only to evaluate the relationship of this species with other congeners. This species is closely related to *A*. *patriciae*, but the phylogenetic relationships of both species within *Austrolebias* are not well supported and contradictory between morphology and morphology+molecular analyses. Previous phylogenetic hypothesis of *Austrolebias* were very unstable in terms of presenting contradictory hypothesis of relationships among species and many unresolved clades or with low support [4, 23, 30], but still some group of species are recovered in the different analyses. These phylogenetic uncertainty could be explained under a sudden explosive speciation process as was previously proposed [23, 31]. Phylogenies hypotheses based on morphological evidence show *Austrolebias patriciae* as the sister group of (*Austrolebias bellottii*+*Austrolebias melanoorus*) species groups [4] or to the *Austrolebias bellottii* species group [30], while an analysis based on *cytb* presented a topology with *A*. *patriciae* as the sister group of the *A*. *alexandri* species group [23]. Morphological evidence indicates that *A*. *varzeae*, from the upper Uruguay basin is related to *A*. *carvalhoi*, from the upper Iguazú basin [4]. Costa [32] proposed the subgenus *Acrolebias* composed of *A*. *varzeae* as type species and *A*. *carvalhoi*, and described *A*. *araucarianus* Costa [24], from the upper Iguazú basin, proposing its inclusion in the *Acrolebias* subgenus, given that: “A clade comprising species of the subgenera *Acrolebias*, *Cypholebias* and *Megalebias* was supported in the last broad phylogenetic analysis of *Austrolebias*” [30], but that phylogeny did not include *A*. *varzeae*. *Sensu* Costa [24] “species of *Acrolebias* are distinguished from species of the subgenera *Cypholebias* and *Megalebias* by having fewer gill-rakers on the ventral portion of the first branchial arch (9 vs. 11–16), fewer neuromasts around the orbit (17–22 vs. 23–38), fewer neuromasts on the otic series (2–3 *vs*. 4–10), and preopercular and mandibular series of neuromasts separated (*vs*. united)”. Here we analyzed those character states for *A*. *wichi* and none of the diagnostic character states of *Acrolebias* are present in this species. Nevertheless, a phylogenetic analysis including the three species proposed for this subgenus has never been performed, hence their monophyly has not been tested yet and the molecular phylogeny by García *et al*. [23] did not include any of these species. Therefore, the use of that subgeneric classification is unjustified from the available evidence and the tentative assignment of *A*. *wichi* to some subgenus of *Austrolebias* is rather nonsensical in this context.

Genetic divergence is frequently used as an indirect inference that two populations belong to separate species, or that several populations belong to a single species [33]. It assumes that isolation is reflected in phenotypic evolution and DNA sequences divergence besides assuming similar evolving rates among species and populations. Nevertheless, these assumptions are not always met, as isolation may not always be reflected in phenotypic divergences or in the other hand, phenotypic divergence among populations can occur with gene flow and may not be reflected at molecular level in particular sequences [34, 35, 36]. Therefore we consider genetic distance, as an additional indirect non-conclusive inference to sustain the new species hypothesis. We used this estimation in order to evaluate the divergence between the new taxon and its closest related species, *A*. *patriciae*. The genetic divergence between the new species and *A*. *patriciae* is around 2%. This value is concordant with previous genetic divergence reported by García *et al*. [37] for interspecies comparisons among species of *Austrolebias* (since 1.5% in closely related species). Generally in fishes, average genetic distance between samples spans for less than 1% within species [38, 39]. The genetic distance estimated support an ancestral population fragmentation from Western and Easter Chacoan regions resulting in the observed divergence between *A*. *wichi* and *A*. *patriciae*.

Also, we find no support for the position of *A*. *varzeae* and *A*. *carvalhoi*, from which we have no molecular data available. Therefore, similar general resemblance of those species color pattern and shape with *A*. *patriciae* and *A*. *wichi*, may be due to plesiomorphic states or to a phylogenetic closeness. Our data does not support neither contradict the monophyly of *Acrolebias* (besides *A*. *araucarianus* was not included in the analysis) as the phylogenetic position of the analyzed species has low support values. Further analyses including additional morphological and molecular data and taxon sampling are needed to clarify the phylogenetic relationship of those species.

Multiple simultaneous speciation seem to have occurred allopatrically in seasonal killifish when Eastern (EC) and Western Chacoan (WC) regions where differentiated. In this area, pairs we observe disjoint distribution of closely related (probable sister) species in each side as *A*. *bellottii andA*. *vandenbergi or A*. *patriciae and A*. *wichi*. This non overlapping distribution among seasonal killifish from WC and EC is observed in other seasonal killifish as *Trigonectes* (*T*. *balzanii* Perugia 1891 in the EC and *T*. *aplocheiloides* Huber 1995 in the WC), *Papiliolebias bitteri* (Costa 1989) in the WC, *Pterolebias longipinnis* Garman 1985 in the EC, [9, 10, 11]. The only seasonal killifish that seems to inhabit both regions is *Neofundulus paraguayensis* (Eigenmann & Kennedy, 1903) although deep morphological analyzes and comparisons with material from the type locality, in the EC, of *N*. *paraguayensis* are still needed to confirm this. Since there are no geographical barriers to the dispersion of these species between western and eastern Chacoan regions, this marked biogeographical separation in annual species distribution seems to be related to ecological constrains and especially to different rainfall regimes [11]. In the WC region, seasonal ponds present water approximately from around December to April (mostly summer) and the next 6 months are dry. On the other hand, in the Eastern Chacoan region the highest precipitations are in autumn and spring resulting in a very contrasting ecological filling/drying cycle [11] and temperatures on which adult fish live.

Also *A*. *elongatus* and *A*. *monstrosus* speciation may be related to this same biogeographic event. Although *A*. *elongatus* is not present in EC but in the pampasic region which seems to be closely related biogeographically to the EC where some species are shared between these regions (as *A*. *nigripinnis* and *A*. *bellottii*). It is possible that *A*. *elongatus* was indeed present in the EC, in most humid and wetter climate times, and it got later locally extinct in this area after weather changes, being restricted nowadays to the southern portion of its original distribution. Alternative explanations for these species current distribution patterns are also plausible.

Colour pattern and general morphology resembles *Austrolebias patriciae*, *Austrolebias varzeae* and *A*. *carvalhoi* (Myers, 1947). It is further distinguished from *A*. *patriciae* by presenting a smaller eye (24.6–29.2 *vs*. 30.1–32.2%HL), a shallower head (69.8–89.4 vs. 108.0–124.3%SL), longer snout (22.9–33.3 vs. 13.9–14.8%HL), and longer jaw (33.2–41.3 *vs*. 19.9–22.0%HL). Additionally, *A*. *wichi* females present shorter caudal fin (23.8–27.7 *vs*. 29.0–29.1%SL), longer head (31.6–35.1 *vs*. 29.4–31.4%SL), dorsal-fin origin anterior to anal-fin origin (*vs*. posterior) and longer incubation period at room temperature of the eggs before hatching than *A*. *patriciae* (6–8 months *vs*. 3–5 months, respectively). *Austrolebias wichi* can be further distinguished from *A*. *patriciae*, *A*. *varzeae*, *carvalhoi*, and *A*. *araucarianus* by presenting different number of gill rakers in first branchial arch (3+11 *vs*. 4+12 in *A*. *patriciae* and 3+9 in the remaining mentioned species). Also, can be differentiated from *A*. *carvalhoi* by having more anal fin rays (23–27 vs. 21–22 in males, and 19–21 *vs*. 16–18 in females) and fewer pectoral-fin rays (10–12 vs. 13), and longer anal-fin base (37.8–44.3 vs. 31.4–35.1%SL). It can be differentiated from *A*. *varzeae* and *A*. *carvalhoi* by a shorter pre-pelvic distance (males: 42.8–49.9, vs. 52.1–55.1 and 52.0–53.2, respectively; females: 47.1–53.7 vs. 54.9–57.8 and 53.9–55.5, respectively; all proportions as %SL), shorter caudal-fin (males: 21.1–27.9 vs. 29.8–34.8 and 30.1, respectively; females: 23.8–27.7 vs. 31.4–35.1 and 30.1, respectively; all proportions as %SL), anal-fin origin between pleural ribs of 7^th^ and 8^th^ vertebrae in males (vs. 9^th^ to 10^th^). Males of *A*. *wichi* present a shallower head than *A*. *patriciae* and *A*. *varzeae* (69.8–89.4 *vs*. 108.0–124.3 and 97.6–107.4%HL, respectively). Females present shorter head than *A*. *patriciae*, *A*. *varzeae* and *A*. *carvalhoi* (70.7–79.3 *vs*. 105.2–105.9; 96.8–101.3; 83.2–97.8%HL, respectively). Males’ body color pattern also differs among these species, which consist of greenish to bluish vertical bands over a pink background and vertical lines of iridescent dots on posterior portion of body in *A*. *wichi* (*vs*. sides of body pale golden with transverse brownish-purple bars without vertical lines of iridescent dots on posterior portion of body in *A*. *carvalhoi*; grey bars over orange background in *A*. *varzeae*; violet grey bands over a whitish background, dorsal and anal fin violet, caudal fin green in *A*. *patriciae*; and light brown background with vertical brown bars in *A*. *araucarianus*).

*Austrolebias wichi* also differs from *A*. *araucarianus* by having pelvic fin and pelvic-fin girdle well developed (*vs*. rudimentary or absent). Differs from the *Austrolebias robustus* species group (*sensu* Costa [4]) by its color pattern, described above vs. males with homogeneous dark gray coloration on flank, sometimes with narrow light lines, but never with bright markings. It can be differentiated from the *A*. *elongatus* species group (*sensu* Costa [4]) by scales present above anal-fin base in females (*vs*. absent) and scales present in anterior portion of frontal region (*vs*. absent). It can be differentiated from the *A*. *alexandri* species group (*sensu* Costa [4]) by pectoral fins hyaline with black border in males (*vs*. dark gray, with bright blue iridescence). *Austrolebias wichi* can be differentiated from the *A*. *bellottii* species group (*sensu* Costa [4]) by having anteromedian anal-fin rays short and fin rounded in females (*vs*. anteromedian rays of anal fin long, resulting in a nearly triangular fin shape). It can be differentiated from the *A*. *adloffi* species group (*sensu* Costa [4]) by the absence of a pair of black spots arranged vertically in close proximity, that sometimes coalesce to form an 8-shaped blotch in the posterior portion of caudal peduncle in juveniles and adult females, sometimes in adult males (*vs*. present).

It can be differentiated from *Austrolebias vandenbergi* (Huber 1995), the other species of the genus present in the Western Chacoan region, by not presenting transverse rows of scales on anal-fin base in males (*vs*. present) and by different colour pattern in males, described before, (*vs*. grey background with vertical grey bands, not always conspicuous in adults).

This new species described here seems to be restricted to a single habitat, despite huge collecting efforts in the area which according to the IUCN criteria put this species under the critically endangered category. This habitat is highly impacted by human activities as soybean plantations that use different chemicals for plague control that are probably affecting this environment. Also, there is evident cattle presence in this paleochannel that alters the pond bottom structure, maybe changing the position at which eggs are and therefore may change the incubation conditions such as humidity and temperatures making development for many eggs non-viable. Taking this into account we consider that this pond and its surroundings should be protected and access to the cattle should be restricted to a limited portion of the pool. Studies on which portion of the pool these species are laying the eggs are planned to be performed in the future.

## Supporting information

S1 Appendix List of material examined. (PDF)

S2 Appendix Character matrix used for the phylogenetic analysis. (PDF)

S3 Appendix Sequences data. (PDF)

S4 Appendix Bayesian Inference (BI) of cyt b sequences. (PDF)
