# Supplementary material for: An endangered new species of seasonal killifish of the genus Austrolebias (Cyprinodontiformes: Aplocheiloidei) from the Bermejo river basin in the Western Chacoan Region
Source: PLoS One. 2018 May 16;13(5):e0196261. doi: 10.1371/journal.pone.0196261 (PMC5955519; doi:10.1371/journal.pone.0196261)
Supplement: S1 Appendix — (PDF) [file pone.0196261.s001.pdf]

Comparative Material

Institutional abbreviations: ANSP, Academy of Natural Sciences, Philadelphia, USA; CI-FML, ichtyological collection Fundación Miguel Lillo, Tucumán, Argentina; FFDU, Grupo Fauna y Flora Dulceacuicola, Montevideo, Uruguay; MACN, Museo Argentino de Ciencias Naturales, Buenos Aires, Argentina; MLP, Museo de La Plata, La Plata, Argentina; MTD-F, Museum für Tierkunde, Dresden, Germany.

Other abbreviations: C&S, cleared and stained;

***Austrolebias alexandri* (Castello & López, 1974).** Argentina: Entre Ríos Province: MACN 6438, holotype, male, 35.3 mm, Parque Unzué, Gualeguaychú; MACN 6439, paratype; 1 female 31.2 mm, same data as holotype. MACN 6425, 4 males 40.6-28.7mm, 3 females 25.4-24.3 mm, same data as holotype. MACN 6437, 4 paratypes: 1 male 30.7 mm, 3 females 28.3-22.7mm, Gualeguaychú; MACN 9696, 27, Ruta 16 near Gualeguaychú; MACN 9736, Ruta 16, 5 km from Ruta 14, Gualeguaychú.

***A. affinis* (Amato 1986).** Uruguay: FFDU 9501, (from type locality) 9 males 21.0-31.0mm and 8 females 18.0-25.0mm, Ruta 5 km 399, 5, Arroyo Tres Cruces, Departamento de Tacuarembó; MTD-F 28135 – 28142, 4 males 34.5-26.5mm, 4 females 24.8-20.7mm, same data as FFDU 9501.

***A. bellottii* (Steindachner, 1881).** Argentina: MACN 8506, 1 male 49 mm, 1 female 38 mm. Temporary pools, 2 km from Estancia "Los Yngleses" and 16 km form Cabo San Antonio, Buenos Aires. aprox. (36°.480 S; 56.820 W); MACN 4278, 3 males, 14 females, San Martín, Buenos Aires; MACN 4279, 3 males, 3 females, Dock Sud, Buenos Aires; MACN 2184, 6 females, Quilmes, Buenos Aires; MACN 2203, 1 male, Vélez Sarfield, Buenos Aires; MACN 497, 1 male, Río Yuquerí Grande, Entre Ríos; MACN 4243, 1 male, Bañado de Flores, Buenos Aires; MACN 1108, 4 males, 3 females, Bañado de Flores, Buenos Aires; MACN 4137, 3 males, 11 females, El Palomar, Buenos Aires; MACN 9687, 10, Brandsen, Buenos Aires; MACN 9708, 15 males, 34 females, Ruta 36 Km 106, Buenos Aires; MACN 9719, 1 female, Ruta 81 Km 1381, Formosa; MACN 9728, 12, Near Pipinas, Buenos Aires; MACN 9739, 10, Gualeguaychú, Entre Ríos; MACN 8506, 1 male 49 mm, 1 female 38 mm, temporary pools 2 km from "estancia Los Yngleses" and 16 km from Cabo San Antonio, Buenos Aires. aprox. 36°.480 S; 56.820 W; MLP 11081, 30, 12-24.4 mm, sewer at 100 m from Guardaparques center, 28º33´24´´S-57º12´31´´W, Laguna Iberá, Esteros del Iberá, Corrientes.

***A. cinereus* (Amato, 1986).** Uruguay: MACN 9704, 3, pools near Arroyo Las Víboras.

***A. nigripinnis* (Regan 1912).** Argentina: MACN 7886, 6, Ruta 12 Km 32, Ituzaingó, Corrientes; MACN 6449, Esteros de Cambán, Corrientes; CI-FML 5171, Garuhapé, Misiones; MACN 8652, 3 males, 5 females, 19.9-24.0 mm, Bañados del río Guaviraví, Yapeyú, Corrientes; MACN 8653, 2 males, 5 females, 27.0-24.5 mm, Bañados del río Aguapey, 5 km before Alvear, Corrientes;

MACN 8654, 1 male, 1 female, 28.8-35.8 mm, Bañados A° Curuzú Cauatia, R 14 km 436, Corrientes; MACN 8655, 2 males, 6 females, 25.1-26.7 mm, near to Médanos, Provincia de Entre Ríos, Argentina; MACN 9688, 4, San Javier, Misiones; MACN 9692, 15, San Javier, Misiones. MACN 9694, 11, Benavidez, Buenos Aires; MACN 9713, 1 male, 6 females, Ibicuycito, Entre Ríos; MACN 9733, 31, Yapeyú, Corrientes; MACN 9734, 1, Magdalena, Buenos Aires; MACN 9738, 34, Gualeguaychú, Entre Ríos.

***A. melanoorus* (Amato, 1986).** Uruguay: MACN 9691, 9, Tacuarembó, Uruguay; MACN 9726, 2, Tacuarembó.

***A. nonoiuliensis* (Taberner, Santos & Castelli, 1975).** Argentina: MACN 6754, paratypes; 12 males, 9 de Julio, Buenos Aires**;** MACN 6755, 17 females, 9 de Julio, Buenos Aires**;** MACN 4241: 1 male, temporary pools near Álzaga, Buenos Aires.

***A. luzardoi* Perujo, Calviño, Salvia & Prieto, 2005.** Uruguay: MACN 9705, 1 male y 7 females, from type locality, Artigas.

***A. robustus* (Günther, 1881).** Argentina: MACN 8507, from type locality, 12 (5 males, 7 females), 49.0-75.0 mm, temporary pools, 2 km from Estancia "Los Yngleses" and 16 km form Cabo San Antonio, Buenos Aires, aprox. 36°.480 S; 56.820 W; MACN 9683, 7, Arroyo La Nutria Mansa, Buenos Aires; MACN 9693, 4, Arroyo La Nutria Mansa, Buenos Aires; MACN 9703, 125 males, 71 females, Arroyo La Nutria Mansa, Buenos Aires; MACN 9716, 40 males, 42 females, Álzaga, Buenos Aires; MACN 9717, 7 males y 8 females, Álzaga, Buenos Aires.

***A. patriciae* (Huber, 1995).** Paraguay: Holotype: ANSP 170424, 1 male, 24.3mm, (pictures and X-ray radiography), ditch along the road to Clorinda, Presidente Hayes Province, about 500 meters south of Río Negro, 25.25°S, 57.67°W; Paratypes: ANSP 173084, 7, same locality as holotype. Argentina: MACN 8876, 5 males, 29.8-33.6 mm, pools near Ruta 11 close to Río de Oro, Chaco, Argentina; MLP 9655, 3 males, 37.6-43.0 mm,9 females, 29.3-36.5 mm, pools near Ruta 11, 400m from Río de Oro, Chaco, Argentina; MACN 9697, 2, pools near Río de Oro, Chaco; MACN 9710, 1 male, 1 female, Puerto Las Plamas, Chaco; MACN 9737, 4, pools near Río de Oro, Chaco.

***A. prognathus* (Amato, 1986).** Uruguay: MACN 9685, 1, Canal Andreoni, Rocha.

***A. toba* Claviño 2006**. Argentina: Holotype: MLP 9652, male, 26.3 mm; temporary pools near Ruta 11, north from Río de Oro, provincia de Chaco. Paratypes: MLP 9653, 9 males 22.7-32.7mm, 9 females 29.3-23.7mm; MACN 8893, 4 males 29.4-35.1mm (C&S); all collected with the holotype.

***A. vandenbergi* (Huber, 1995).** Argentina: MACN 9701, 26, Padre Lozano, Salta; MACN 9707, 19, Padre Lozano, Salta; MACN 9718, 5, Laguna Yema, Formosa; CI- FML 5318, 14, 5 km west from El Vizcacheral, General Güemes, Chaco, 24º23'29''S, 61º45'50''W; CI-FML 5322, 3, road to Güemes, 1 km north from Río Bermejito, General Güemes, Chaco 24º46'06.8''S, 61º48'

30.1’’W; CI-FML 5324, 13, same data as CI-FML 5322; CI-FML 5340, 3, same data as CI-FML 5322; CI- FML 5337, 2, 5 km west to El Vizcacheral, General Güemes, Chaco, Argentina, 24º23’29’’S, 61º45’50’’W; CI-FML 5341, 6, El Sauzalito, near to Paraje Zanjas, General Güemes, Chaco, Argentina, 24º29’26.8’’S, 61º48’33.4’’W.

***A. vazferreirai* (Berkenkamp, Etzel, Reichert & Salvia, 1994)**. Uruguay: MACN 9690, 9, ponds near Río Negro; MACN 9732, 3, ponds near Río Negro.

*** Additional comparative material listed in Calviño *et al.* (2016) and Alonso *et al.* (2016).**

References

Alonso, F., Calviño, P. A., Terán, G. E., & García, I. (2016). Geographical distribution of *Austrolebias monstrosus* (Huber, 1995), *A. elongatus* (Steindachner, 1881) and *A. vandenbergi* (Huber, 1995)(Teleostei: Cyprinodontiformes), with comments on the biogeography and ecology of Rivulidae in Pampasic and Chaco floodplains. Check List, 12(4), 1945.

Calviño, P. Nadalin D. O.; Ma. J. Serio M. J. & H. L. López. 2016. Colección Ictiológica del Museo de La Plata: la familia Rivulidae. Probiota, FCNyM, UNLP, La Plata, Argentina, serie Técnica y Didáctica 36: 1-21.
