# Supplementary material for: An endangered new species of seasonal killifish of the genus Austrolebias (Cyprinodontiformes: Aplocheiloidei) from the Bermejo river basin in the Western Chacoan Region
Source: PLoS One. 2018 May 16;13(5):e0196261. doi: 10.1371/journal.pone.0196261 (PMC5955519; doi:10.1371/journal.pone.0196261)
Supplement: S2 Appendix — (PDF) [file pone.0196261.s002.pdf]

```
nstates dna
xread
936 40
& [num]
N_paraguayensis 000000000000010000100?0000000000000000000000000000000000000000000?0000000000
N_whitei 00100001200000000000?00020100000000000000000001000100001?00000100000100?0000000011
S_bokermanni 0010000110000000000010000101000000000001000000200010000000000001011011100?0000000010
C_griseus 110000011000000000?3000010121003202010100000121111000000000000101101100?0000000010
A_adloffi 0212111221000000100211000101000020020101001001201111101000000010111112020101010100002
A_affinis 011011022100000010041000010100002002010100100120111100100000001011111202010010000011
A_alexandri 011011022100000010041000010100001002010100100120011100100000001011111202010010010 10
A_arachan 01111112210000001001110001010000200201010010012011111010001000111111120201000100010
A_bellottii 121211122110000010031100010100002001010100100120111110101010001111111202010001100 10
A_carvalhoi 0100110221000000100310000101000010020101001001201111101000000010111112020100000000 10
A_charrua 02111112210000001002110001010000200201010010012011111010000000101111120201000100012
A_cheradophilus 10001101111000031222?302003011001201211110010012111100011?0011011111112120100000000 0
A_cinereus 011111022100000010023020020100002002010100100120111110100110011011111202011000000 10
A_cyaneus 011011022100000010041000010100000002010100100120111100100000001011111202010010000011
A_duraznensis 0110110221000000100410000101000010020101001001201111001000000010111112020100100000 11
A_elongatus 211111011101111011 2?302113122111311211111211112111100120000110121111121201000000000
A_gymnoventris 01101103210000012001200001010000000201010010012011101010000000101111120100100000000 0
A_ibicuiensis 0110110221000000100410000101000010020101001001200111001000000010111112020100100101 0
A_jaegari 01101103210000012001200001010000000201010010012011111010000000101111120100100000000
A_juanlangi 0110110221000000100410000101000000020101001001201111001000000010111112020100100000 11
A_litzi 011011022100000010041000010100000002010100100120111100100000001011111202010010000011
A_luteoflammulatus 01111103210000001001201002010000000201010010012011100010000000101111120201000000100
A_melanoorus 01111112211000001004 0?0001011000200101010010012011111010001000111111120201000110010
A_minuano 02111112210000001002110001010000200201010010012011111010000000101111120201010100012
A_monstrosus 211111011101111011 2?302113122111311211111211112111100120000110121111121201100000000
A_nachtigalli 02111112210000001003110001010000200201010010012011111010000000101111120201010100102
A_nigripinnis 01111102210000001004100001010000100201010010012001100010000000101111120211001000000
A_nigrofasciatus 0211111221000000100211000101000020020101001001201111101000000010111112020101010010 2
A_paranaensis 01111102210000001004100001010000100201010010012001110010000000101111120211001000000
A_patriciae 01111102210000001004100001010000100201010010012011111010000000111111120201000100021
A_periodicus 01101102210000001004100001010000000201010010012011110010000000101111120201001000011
A_prognathus 20111101110100101 12?302113122111321211111211112111100120000110121111121201000000000
A_robustus 11111101110000001012302002010000200201010010012011111010010001101111120201100000003
A_univentripinnis 01121112211000001003110001010000200101010010012011111010001000111111120201000110010
```

A_vandenbergi 121211122101000010143120020110002001010100100120111110101010001211111202010001000100
A_varzeae 01001102210000001002100001010000100201010010012011111010000000101111120201000000010
A_vazferreirai 1111110221000000101230200201000020020101001001201111101001000110111112020110000011
A_viarius 02111112210000001002110001010000200201010010012011111010001000111111120201000000010
A_wolterstorffi 1000110111000020111?20000102210130121111111001211110012100011010111112120100000000
A_wichi 0[01]1111022100000010041010000100001002010100100 12?111110100000001[01]11111202010001100 21

& [dna]
A_nigripinnis
????????????????????????????????AATGGCCCCCTACGAAAAACTCACCCTCTTTTTAAACTGTTTAATAGCGCTTTAGTAGATCTTCCTACACCTCCCAAT
ATCTCATCATGATGAAACTTTGGCTCTTTATTAGGCTTATGCTTAGCCACTCAAATTTTTACTGGGCTTTTTTTAGCAATACATTACACCCCCGACACAATAATAGCTTTT
TCTTCCATCGCACATATTTGCCGTGATGTAAATTACGGGTGGCTTATTCGTAACATTCATGCAAATGGAGCTTCTTTCTTTTTTATCTGTCTTTACGCCCACATTACCCGC
GGTCTTTATTATGGTTCTTACTTGCATAAAGAAACATGAAATGTTGGCGTAATTCTTTTACTCCTTGTAATAATAACTGCTTTCGTCGGTTACGTTCTTCCCTGAGGCCAA
ATATCGTTCTGGGGGGCAACTGTAATCACAAACCTTCTCTCCGCTATCCCCTTTTTAGGAGAAGCTCTAGTACAATGAATCTGGGGGGGATTTCAGTAGATAATGCAACC
CTCACTCGATTTTTTGCTTTTCACTTCCTGCTGCCTTTTGTAGTAATAGCTTTAGTTATAATTCATCTAATTTTCTTACATGAAACCGGCTCTAACAACCCAGCAGGCCTT
AACTCAAATGCAGAAAAAATTTCATTCCACCCTTACTTCTCGTATAAAGATTTTTTTGGTTTCACATTATTCTTCCTGCTCCTTATAATCCTCGCCCTCTTTTCTCCAAAC
CTTCTCGGAGATCCGGAAAACTTTTCGTCAGCCAACCCTTTAATAANNNNNNNNN????????????????????
A_cheradophilus
????????????????????????????????AATGGCCCCCTACGAAAAACTCATCCCCTTTTTAAATTACTCAATAGTGCTTTAGTAGACCTTCCCACTCCTCCAAAC
ATTTCATCGTGATGAAACTTTGGTTCTTTACTTGGCCTCTGTCTAGCAACACAGATCTTAACCGGGCTTTTTTTAGCAATGCACTATACCCCTGACACTTCAATAGCTTTT
TCCTCCGTAGCCCACATTTGTCGCGATGTCAACTACGGGTGACTCATTCGCAATATTCATGCAAACGGAGCTTCCCTTTTCTTCATCTGTCTCTATGCACACATTACCCGA
GGCCTTTACTATGGCTCTTACTTATATAAAGAGACCTGAAATACAGGCGTCGTAATTTTACTTTTAGTAATAATAACAGCCTTCGTAGGCTACGTTCTTCCCTGAGGCCAA
ATGTCCTTTTGGGGTGCAACAGTTATTACCAACNNNNNNNNNNNNNNNNNNNNNNNNNNNNNNNNNNNNNNNNNNNNNNNNNNNNNNNNNNNNNNNNNNNNNNNNNNNNN
NNNNNNNNNNNNNNNNNNNNNNNNNNNNNNNNNNNNNNNNNNNNNNNNNNNNNNNNNNNNNNNNNNNNNNNNNNNNNNNNNNNNNNNNNNNNNNNNNNNNNNNNNNNNNNN
NNNNNNNNNNNNNNNNNNNNNNNNNNNNNNNNNNNNNNNNNNNNNNNNNNNNNNNNNNNNNNNNNNNNNNNNNNNNNNNNNNNNNNNNNNNNNNNNNNNNNNNNNNNNNNN
NNNNNNNNNNNNNNNNNNNNNNNNNNNNNNNNNNNNNNNNNNNNNNNNNNNNNNN????????????????????
A_duraznensis
????????????????????????????????AATGGCCCCCTACGAAAAACCCACCCCCTTTTTAAATTACTAAATAATGCTTTAGTAGACCTCCCTACCCCTCCCAAT
ATTTCATCATGGTGAAATATGGGCTCTTTATTAGGACTATGTTTAGCGACTCAAATCTTAACCGGGCTTTTTTTAGCAATACATTATACGCCTGACACCACAATAGCATTC
TCTTCTGTTGCTCACATTTGTCGTGATGTTAATTACGGTTGACTCATTCGAAATATTCATGCAAACGGAGCCTCCTTTTTTTTTCTTTGTCTTTATGCTCATATTACTCGA
GGCCTTTACTATGGCTCTTACTTGTACAAAATAACATGAAACACTGGTGTAGTTATTTTGCTCCTTGTAATAATAACCGCTTTTGTTGGTTATGTTCTTCCTTGAGGTCAA
ATGTCTTTTTGAGGTGCTACAGTAATTACTAATCTCCTTTCCGCTATCCCCTTTTTAGGAGAAACCCTTGTGCAATGAATTTGAGGGGGTTTTTCAGTAGACAATGCAACC
CTTACCCGATTTTTTGCATTCCATTTCCTACTTCCCTTTGTAGTAGCAGCTATAGTTCTCGTACACTTAATTTTCCTTCATGAAACGGGCTCCAACAACCCAGCAGGCCTA
AATTCAAACACAGACAAAGTCTCCTTTCATCCCTACTTTTCCTATAAAGACCTTTTTGGTTTTGCACTATTTTTTCTTCTGCTTATATATCTTGTCTTATTTTATCCAAAT
ATTCTAGGAGACCCCGAAAATTTCTCGCCTGCAAACCCTCTAATAANNNNNNNNN????????????????????
A_alexandri
????????????????????????????????AATGGCCCCTTACGAAAGAACCACCCCCTTTTTAAATTATTTAACGATGCTTTAGTAGACCTCCCCACCCCCCCCAAT
ATTTCATCATGATGAAATATAGGCTCTTTATTAGGCCTATGTTTAGCAACCCAGATCTTAACTGGACTCTTTTTAGCGATACACTACACACCTGATACCACAATAGCATTC

TCTTCTGTTGCTCACATTTGTCGTGATGTTAATTACGGCTGACTCATTCGTAACATTCATGCAAATGGGGCTTCCTTTTTTTTTATTTGTCTCTATGCTCACATCACTCGC
GGCCTTTACTATGGCTCTTACTTATATAAAATAACATGGAACACTGGGGTCATCATTTTGCTCCTTGTAATGATAACTGCTTTTGTTGGTTACGTTCTTCCTTGAGGACAA
ATATCTTTTTGGGGCGCTACAGTAATTACTAACCTCCTTTCCGCAATCCCCTTTTTAGGAGAAATTCTCGTGCAATGAATCTGAGGGGGCTTCTCAGTAGATAACGCAACC
CTTACCCGATTTTTTGCATTCCACTTTCTACTTCCCTTTGTAGTAGCAGCCATAGTTCTTGTGCACTTAATTTTTCTTCATGAGACCGGCTCCAACAACCCAGCAGGCCTA
AATTCAAACTCAGATAAAATTTCATTTCATCCATACTTTTTGTATAAAGACCTTTTTGGTTTTGGGCTATTTTTTCTTTTTCTTATACTACTTGTCTTATTTAACCCAAAC
ACTTTAGGGGATCCGGAAAATTTCTCGCCTGCAAATCCTCTAATAANNNNNNNNN?????????????????????
A_wolterstorffi
?????????????????????????????????TGGCCCCAATTACGAAAAACTCATCCTCTTTTTAAATTATTCAACGATGCTTTAGTAGACCTTCCTACTCCTCCCAAT
ATTTCATCATGATGAAATATTGGTTCTCTATTAGGTCTTTGCTTAGCAACACAAATCTTAACTGGACTTTTTTTAGCAATACATTATACTCCTGATACCACGATAGCTTTT
TCTTCTGTGGCTCATATTTGTCGCGATGTAAACTACGGGTGACTCATCCGAAATATTCATGCAAACGGAGCCTCTCTCTTTTTTTATCTGTCTCTATGCTCATATTACCCGC
GGTCTCTACTATGGCTCTTACTTATACAAAGAAACGTGAAATACAGGAGTTCTAATTTTGCTTCTAGTAATAATAACTGCCTTTGTAGGCTACGTCCTTCCTTGAGGTCAG
ATGTCTTTTTGAGGGGCTACAGTAATTACCAACCTTCTTTCCGCCATCCCATTTTTAGGGGAAACTCTCGTGCAATGAATTTGAGGGGGATTTTCAGTAGACAACGCAACC
CTCACCCGATTCTTCGCGTTTCATTTCTTACTGCCTTTTGTAGTAGCAGCCATAGTCCTTATCCACCTTATCTTTCTCCATGAGACAGGTTCAAATAACCCCGTCGGGCTA
AATTCAAACACAGACAAAATTATATTCCACCCTTACTTTACGTACAAAGACCTTTTTGGTTTTATATTATTCTTCCTCCTCCTTACAATATTTGCTCTCTTCTATCCCAAT
CTTCTTGGAGACCCAGAAAATTTTACCCCTGCTAACCCCCTAATAANNNNNNNNN?????????????????????
A_arachan
?????????????????????????????????NNNNNNNNNNNNNNNNNNNNNNNNNNNNNNNNNNNNNNNNNNNNNNNNNNNNNNNNNNNNNNNNNNNNNNNNNNNNNNNNNNNNT
ATCTCATTATGGTGAAACTTTGGTTCTTTACTAGGCCTCTGTTTAATAGTACAAATCTTAACTGGGCTCTTTTTAGCAATACATTATACCCCCGACACCATAATAGCCTTT
TCTTCTGTTGCCCATATTTGCCGCGATGTAAATTACGGCTGACTTATTCACAATCTCCATGCAAATGGCGCTTCTTTCTTTTTTATTTGCCTTTATGCTCATATTACTCAA
GGCCTTTACTATGGTTCTTACCTGTATAAAGAAACATGAAACACGGGGGTCTTTATTTTACTTCTTGTAATGATGACTGCTTTTGTAGGCTNNNNNNNNNNNNNNNNNNNN
NNNNNNNNNNNNNNNNNNNNNNNNNNNNNNNNNNNNNNNNNNNNNNNNNNNNNNNNNNNNNNNNNNNNNNNNNNNNNNNNNNNNNNNNNNNNNNNNNNNNNNNNNNNNNNNNN
NNNNNNNNNNNNNNNNNNNNNNNNNNNNNNNNNNNNNNNNNNNNNNNNNNNNNNNNNNNNNNNNNNNNNNNNNNNNNNNNNNNNNNNNNNNNNCAAATAATCCAGTAGGCCTA
AATTCAAATGCGGATAAAATTTCATTCCACCCTTACTTCTTATATAAAGACTTGTTTGGTTTTATACNNNNNNNNNNNNNNNNNNNNNNNNNNNNNNNNNNNNNNNNNNNNNNN
NNNNNNNNNNNNNNNNNNNNNNNNNNNNNNNNNNNNNNNNNNNNNNNNNNNNNNN?????????????????????
A_vazferreirai
????????????????????????????????AATGGCCCCT?TACGAAAAATNCATCCTCTTTTTAAATTACTTAACAGCGCTTTAGTAGACCTTCCCACCCCCCCTAAT
ATCTCATCATGATGAAACTTTGGCTCTCTACTAGGCCTCTGTTTAGCAACGCAAATCTTAACTGGACTTTTTTTAGCCATACACTATACTCCTGACACCACTATAGCTTTT
TCTTCTGTAGCTCATATTTGTCGTGATGTAAATTACGGTTGGCTCATTCGTAGTATTCATGCAAATGGGGCTTCTCTTTTTTTCATTTGCCTTTATGCTCATATTACCCGA
GGCCTTTACTACGGCTCTTACTTATATAAANAAACATGAAATACCGGGGTTATAATCTTGCTTCTAGTAATGATAACTGCTTTTGTAGGCTACGTTCTTCCTTGAGGACAG
ATGTCCTTTTGGGGAGCAACAGTTATTACTAACCTTCTTTCTGCAATCCCTTATTTAGGAGAAACCCTTGTTCAATGAATCTGGGGGGGCTTTTCAGTAAATAATGCGACT
TTAACCCGATTCTTTGCATTTCACTTTTTGCTTCCCTTTGTAGTGGCAGCTGTAGTCATGGTCCACTTAATTTTTCTACATGAAACTGGCTCAAATAACCCAGCAGGGCTA
AATTCAAACACAGACAAAATTTCCTTCCACCCTTATTTTACTTATAAAGACCTTTTTGGGTTTATACTATTCTTTCTGACCCTCACAATGCTTGCTCTTTTTTACCCCAAT
CTCCTCGGANACCCANAAAATTTTCCCCTTNNNNNNNNNNNNNNNNNNNNNNNNN?????????????????????
A_cinereus
????????????????????????????????AATGGCCCCT?TACGAAAAATCATCCNTCTTTTTAAATTACTTAACAGCGCTTTAGTAGACCTTCCCACCCCCCCTAAT
ATCTCATCATGATGAAACTTTGGCTCTCTACTAGGCCTCTGTTTAGCAACGCAAATCTTAACTGGACTTTTTTTAGCCATACACTATACTCCTGACACCACTATAGCTTTT
TCTTCTGTGGCTCATATTTGTCGTGATGTAAATTACGGTTGGCTCATTCGTAGTATTCATGCAAATGGGGCTTCTCTTTTTTTCATTTGCCTTTATGCTCATATTACCCGA
GGCCTTTACTACGGCTCTTACTTATATAAAGAAACATGAAATACCGGGGTTATAATCTTGCTCCTAGTAATGATAACTGCTTTTGTAGGCTACGTTCTTCCTTGAGGACAN

ATGTCCTTTTGGGGGGCAACAGTTATTACTAACCTTCTTTCTGCAATCCCTTATTTAGGAGAAACCCTTGTTCAATGAATCTGGGGGGGCTTTTCAGTAGATAATGCGACT
TTACCCGATTTCTTTGCATTTCACTTTTTGCTTCCCTTTGTAGTGGCACCTGTAGTCATGGTCCACTTAATTTTTCTACGTGAAACTGGCTCAAATAACCCAGCAGGCCTA
AATTCAACACAAGACAAAATTTCCTTCCACCCTTATTTTACTTATAAAGAACTTTTTGGGGTTATACAATTCTTTCTTGATCTCACAATGCTTGNCCTTTTTTACCCAAAT
CTCCTCGGAAAACCAAAAAATTTTTCACCTGNCAACCCNNNNNNNNNNNNNNNNN?????????????????????
A_melanoorus
?????????????????????????????????NNNNNNNNNNNNNNNNNNNNNNNNNNNNNNNNNNNNNNNNNNNNNNNNNNNNNNNNNNNNNNNNNNNNNNNNNNNNNNNN
NNNNNNNNNNNNNNNNNNNNNNNNNNNNNNNNNNNNNNNNNNNNNNNNNNNNNNNNNNNACGGGGACTTTTTTTAGCCATACACTACACTCCGGACACTGCGATGGCTTTT
TCTTCTGTTGCTCATATTTGTCGCGATGTAAATTACGGGTGGTTCATCGGTAATATACATGCAAATGGGGCTTCTCTTTTTTTTCATTTGCCTCTATGCCCACATTACCCCA
GGGCCTTACTACGGCTCCTACTTATATAAAGAAACATGAAATACCGGGGTTGTAATCTTGCTTCTAGTAATGATAACTCCTTTTGTAGGTTATGTTCTTCCTTGAGGGCAA
ATGTCCTTTTGAGGGGCAACAGTTATTACTAACCTTCTTTCTGCGATTCCTTATTTAGGAGAAACCCTTGTTCAATGAATTTGGGGGGGCTTTTCAGTAAATAATGCGACT
TTAACTCGATTCTTTGCATTTCACTTTTTGCTTCCCTTTGTAGTGGCAGCTGTAGTCATGGTCCACTTAATTTTTCTACATGAAACTGGCTCAAATAATCCAGCAGGCCTA
AATTCAAGCACAGATAAAATTTCCTTCCACCCTTACTTTACTTATAAAGACCTTTTTGGCTTCATACTATTCTTCCTTGTTCTCGCAACACTTGCTCTTTTTTACCCTAAT
CTTCTAGGTGATCCAGAGAACTTTTCCCCCGCCAATCCTTTAATANNNNNNNNNNN?????????????????????
A_juanlangi
?????????????????????????????????NNNNNNNNNTACGAAAAACCCACCCCCTTTTTAAATTACTAAATAATGCTTTAGTAGATCTCCCTACCCCTCCCAAT
ATTTCATCGTGGTGAAATATGGGCTCTTTATTAGGCCTATGTTTAGCTACTCAAATCTTAACTGGGCTTTTTTTAGCAATACATTATACGCCCGACACCACAATAGCATTC
TCTTCTGTTGCTCACATTTGTCGTGATGTTAATTATGGTTGACTCATTCGAAACATTCATGCAAATGGGGCCTCCTTTTTTTTTCTTTGTCTTTATGCTCACATTACTCGA
GGCCTTTACTATGGCTCTTACTTATACAAAGTAACATGAAATACTGGTGTAGCTATTTTGCTCCTTGTAATAATAACCGCTTTTGTANNNNNNNNNNNNNNNNNNNNNNNN
NNNNNNNNNNNNNNNNNNNNNNNNNNNNNNNNNNNNNNNNNNNNNNNNNNNNNNNNNNNNNNNNNNNNNNNNNNNNNNNNNNNNNNNNNNNNNNNNNNNNNNNNNNNNNNNNN
NNNNNNNNNNNNNNNNNNNNNNNNNNNNNNNNNNNNNNNNNNNNNNNNNNNNNNNNNNNNNNNNNNNNNNNNNNNNNNNNTGAAACGGGCTCCAACAACCTAGCAGGCCTA
AATTCAAATACAGATAAAATCTCCTTTCATCCCTACTTTTNATATAAAGACCTTTTTGGTTTTACACTATTTTTTCTTCTCCTTATATATCTTGTCTTATTTTATCCAAAT
ATTCTAGGAGACCCTGAAAATTTCTCGCCTGCAAACCCTTTAATAANNNNNNNNNN?????????????????????
A_patriciae
?????????????????????????????????NNNNNNNNNNNNNNNNNNNNNNNNNNNNNNNNNNNNNNNNNNNNNNNNNNNNNNNNNNNNNNNNNNNNNNNNNNNNNNNN
NNNNCCTGGGGATGAAACTTTGGCTCTCTACTAGGCCTCTGTTTAGCAACACAAGTTTTAACAGGACTTTTTTTAGCAATACACTACACCCCTGACACTACAATAGCTTTT
TCATCAGTAATTCATATTGGTCGTGATGTAAATTATGGATGACTCATCCGAAATATTCATGCAAACGGAGCTTCATTCTTTTTTATTTGTCTATACGCTCATGTTTCTCGA
GGGCTTTATTATGGTTCTTATTTATATAAAATAACATGAAACACTGGAGTTGTTATTTTACTCCTTGTAATAATAACTGCCTTTGTAGGGTACGTTCTTCCCTGAGGCCAA
ATGTCTTTTTGAGGGGCAACAGTTATTACTAATCTTCTTTCCGCAATCCCTTTTTTAGGAGAAANTTTATTTCNATGAATTTGAGGTGGNNNNTCNGTAGATAATGCAACC
CTTACCCGATTNNNNNNNNNNNNNNNNNNNNNNNNNNNNNNNNNNNNNNNNNNNNNNNNNNNNNNNNNNNNNNNNNNNNNNNNNNNNNNNNNNNNNNNNNNNNNNNNNNNNNN
NNNNNNNNNNNNNNNNNNNNNNNNNNNNNNNNNNNNNNNNNNNNNNNNNNNNNNNNNNNNNNNNNNNNNNNNNNNNNNNNNNNNNNNNNNNNNNNNNNNNNNNNNNNNNNNNN
NNNNNNNNNNNNNNNNNNNNNNNNNNNNNNNNNNNNNNNNNNNNNNNNNNNNNNNNN?????????????????????
A_vandenbergi
?????????????????????????????????NNNNNNNNNNNNNNNNNNNNNNNNNNNNNNNNNNNNNNNNNNNNNNNNNNNNNNNNNNNNNNNNNNNNNNNNNNNNNNNN
NNNNNNTCATGATGAAATTTTGGCTCTTTACTAGGCCTCTGTCTAGCCGTACAAATCTTAACTGGGCTTTTTCTAGCAATACATTATACACCGGACACTACTATAGCTTTT
TCTTCTGTTGCTAATATTTGTCGCGATGTAAACTACGGATGGCTTATTCGTAATATTCATGCAAATGGGGCCTTTCTTTTTTTCATTTGTCTTTATGCACACATCACTCGA
GGTCTTTACTACGGCTCTTACTTGTATAAAGAAACATGAAACACCGGAGTCTTAATCTTACTTTTAGTAATAATAACTGCTTTTGTAGGTTATGTTCTCCCTTGAGGGCAA
ATATCCTTCTGAGGTGCAACAGTTATTACTAATCTTCTNTCCGCAATTCCGTTTTTAGGAGAAACCTTTGTTCAATGAATTTGAGGGGGATTTTCCGTAGACAACGCAACC
TTAACCCGTTTTTTCGCATTTCANNNNNNNNNNNNNNNNNNNNNNNNNNNNNNNNNNNNNNNNNNNNNNNNNNNNNNNNNNNNNNNNNNNNNNNNNNNNNNNNNNNNNNNNNN

NNNNNNNNNNNNNNNNNNNNNNNNNNNNNNNNNNNNNNNNNNNNNNNNNNNNNNNNNNNNNNNNNNNNNNNNNNNNNNNNNNNNNNNNNNNNNNNNNNNNNNNNNNNNNN
NNNNNNNNNNNNNNNNNNNNNNNNNNNNNNNNNNNNNNNNNNNNNNNNNNNNNNN?????????????????????
A_monstrosus
????????????????????????????????AATGGCCCCCTACGAAAAGCTCACCCCCTTTTTAAGTTATTTAATAGTGCCTTAGTAGACCTTCCAACTCCTCCTAAT
ATTTCATCATGGTGAAACTTTGGCTCTTTACTTGGCCTCTGCTTAGCAACACAGCTCTTAACTGGGCTTTTTTAGCAATGCACTATACCCCGGATACTACAATAGCTTTT
TCTTCTGTAGCCCACATTTGTCGCGACGTTAACTACGGATGGCTTATTCGTAATATTCATGCAAATGGAGCTTCTCTTTTTTTCATCTGTCTTTATGCTCACATCACCCGA
GGCCTCTACTATGGTTCTTACTTATATAAAGAAACGTGAAATACAGGTGTCGTAATTTTACTTCTAGTAATAATAACGGCCTTTGTAGGCTACGTTCTTCCTTGAGGCCAA
ATATCTTTTTGAGGAGCTACAGTTATTACTAATCTTCTTTCCGCAATCCCTTTTTTAGGTGAAACTCTTGTTCAATGAATTTGAGGGGGGTTTTCAGTAGATAACGCAACC
TTAACCCGATTCTTTGCGTTTCATTTCTTACTGCCTTTTGTTGTAGCAGCTATAGTCATAATTCACCTAATTTTCCTTCATGAATCTGGTTCTAATAATCCCGTAAACCNN
NNNNNNNNNNNNNNNNNNNNNNNNNNNNNNNNNNNNNNNNNNNNNNNNNNNNNNNNNNNNNNNNNNNNNNNNNNNNNNNNNNNNNNNNNNNNNNNNNNNNNNNNNNNNNN
NNNNNNNNNNNNNNNNNNNNNNNNNNNNNNNNNNNNNNNNNNNNNNNNNNNNNNN?????????????????????
A_periodicus
????????????????????????????????NNNNNNNNNNNNNNNNNNNNNNNNNNNNNNNNNNNNNNNNNNNNNNNNNNNNNNNNNNNNNNNNNNNNNNNNNNNNNN
NNNNNNNNNNNNNNNNNNNNNNNNNNNNNNNNNNNNNNNNNNNNNNNNNNNNNNNNNNNNNNNNNNNNNNNNNNNNNNNNNNNNNNNNNNNNNNNNNNNNNNNNNNNNNN
NNNNNNNNNNNNNNNNNNNNNNNNNNNNNNNNNNNNNNNNNNNNNNNNNNNNNNNNNNNNNNNNNNNNNNNNNNNNNNNNNNNNNNNNNNNNNNNNNNNNNNNNNNNNNN
NNNNNTTACTATGGCTCTTACTTATACAAAGTAACATGAAATACTGGTGTAGCTATTTTGCTCCTTGTAATAATAACCGCTTTTGTTGGTTATGTTCTTCCTTGAGGTCAA
ATGTCTTTTTGAGGTGCTACAGTAATTACTAATCTCCTCTCCGCTATCCCCTTTTTAGGAGAAACCCTCGTGCAATGAATTTGAGGTGGGTTTTCAGTAGATAATGCAACT
CTTACCCGATTTTTTGCATTCCATTTCATACTTCCCTTTGTAGTAGCAGCTATAGTTCTCGTACACTTAATTTTCCTTCATGAAACGGCTCCCAACAACCCAGCAGGCCTA
AATTCAAACTCAGATAAAATCTCATTTCATCCCTACTTTTCATATAAAGACCTTTTTGGTTTTGCACTATTTTTTCTTCTCCTTATACTACTTGTCTTATTTAATCCAAAT
ACTTTAGGGGATCCGGAAAATTCCTCGCCTGCAAATCCTCTAATAANNNNNNNNN?????????????????????
A_cyaneus
??TTTAAATTATTCAACGATGCTTTAGTAGACCTCCCTACTCCCCCCAAT
ATTTCATCATGATGAAATATGGGTTCTTTATTAGGCCTATGCTTAGCAACTCAGGTCTTAACTGGACTTTTTCTAGCAATACACTATACACCGGACACCACAATAGCATTT
TCTTCTGTCGCTCACATTTGTCGTGACGTTAATTACGGCTGACTCATTCGAAATATTCATGCAAACGGAGCTTCCTTTTTTTTTCTTTNTCTTTACNCTCACATTACTCNA
GGCCTTTACTATGGCTCTTACTTGTATAAAGTAACATGAAACACTGGTGTCATTATCTTACTCCTTGTAATAATAACCNCTTTTGTTGGTTACGTTCTCCCCTGAGGACAA
ATATCTTTTTGAGGCGCTACAGTAATTACTAATCTCCTTTCCGCTATTCCCTTTTTAGGAGAAAA???
??
??
??
A_minuano
????????????????????????????????ATGGCCCCCTTACGAAAAACTCACCCCCTTCTTAAGTTATTTAATAGTGCTTTAGTAGACCTTCCTACTCCTCCTAAT
ATCTCATTATGATGAAACTTTGGCTCTTTATTAGGCCTTTGTCTAGCAACACAGATCTTAACCGGGCTTTTTTAGCAATACATTATACTCCTGACACCACAATAGCTTTT
TCTTCTGTCGCCCATATTTGTCGCGATGTAAACTACGGGTGACTTATTCGCAATGTTCATGCAAATGGCGCTTCTTTCTTTTTTATTTGTCTTTATGCTCATATTACTCGA
GGCCTTTACTATGGCTCCTACCTGTACAAAGAAACATGAAACACCGGGGTCGTTATTTTACTTCTCGTAATGATGACTGCTTTTGTAGGCTACGTTCTTCCTTGAGGGCAG
ATATCTTTTTGGGGGGCAACAGTCATTACGAACCTTCTTTCCGCAATTCCTTTTTTAGGGGAAACTCTTGTACAATGAATTTGAGGGGGTTTCTCAGTAGATAATGCAACT
CTAACCCGATTTTTTGCATTCCATTTCTTATTTCCCTTTGTGGTAATAGCCATAGTTATAATTCACCTAATTTTCCTTCATGAAACTGGCTCTAATAATCCGGTAGGCCTA
AATTCAAATGCAGATAAAATTTCATTCCACCCTTACTTCTTATACAAAGACCTATTTGGTTTCATACTATTTTTTGCCCTTCTCATAATATTTGCTCTTTTTTACCCTAAT
CTTCTAGGTGACCCAGAAAATTTTTCCCCCGCCAACCCCCTAATAACACCCCCTCATATTAAGCCCGAATGATACT

A_nachtigalli
????????????????????????????????ATGGCCCCCCTACGAAAAACTCACCCCCTTCTTAAGTTAATTAATACTGCTTTAGTAGACCTTCCTACTCCTCCTAAT
ATCTCATTATGATGAAACTTTGGCTCTTTATTAGGCCTTTGTCTAATAATACAGATCTTAACCGGGCTTTTTTAGCAATACATTACACTCCTGACACCACAATAGCTTTT
TCTTCTGTCGCCCATATTTGTCGCGATGTAAACTACGGGTGACTTATTCGCAATATTCATGCAAATGGCGCTTCTTTCTTTTTTATTTGTCTTTATGCTCATATTACTCGA
GGCCTTTACTATGGCTCCTACCTGTACAAAGAAACATGAAATACGGGGGTCGTTATTTTACTTCTCGTAATGATGACTGCTTTTGTAGGCTACGTTCTCCCTTGAGGGCAG
ATATCTTTTTGGGGGGCAACAGTCATTACGAACCTTCTTTCCGCAATTCCTTTTTTAGGGGAAACTCTTGTACAATGAATTTGAGGGGGTTTCTCAGTAGATAATGCAACT
CTAACCCGATTTTTTGCCTTTCATTTCTTATTCCCCTTTGTGGTAATAGCCATAGTTATAATTCACCTAATTTTTCTTCATGAGACTGGCTCTAACAATCCAGTAGGCCTA
AATTCAAATGCAGATAAAATTTCCTTCCACCCTTACTTCTCATACAAGGACCTATTTGGTTTCATACTATTTTTTGTTATTCTCATAATATTTGCTCTTTTTTATCCCAAC
CTTCTAGGTGACCCAGAAAATTTTTCCCCCGCCAACCCCCTAATAACGCCCCCTCATATTAAGCCCGAATGA????
A_nigrofasciatus
????????????????????????????????ATGGCCCCCCTACGAAAAACCCACCCCCTTTTTAAATTACTTAATAGTGCTCTAGTAGACCTTCCTGCTCCCCCTAAC
ATCTCATTATGATGAAACTTTGGCTCTTTACTAGGTCTATGCTTAGTAACACAAATCTTAACTGGACTTTTTTAGCAATACATTACACCCCTGACACCACGATAGCTTTT
TCTTCTGTAGCTCATATTTGTCGCGATGTAAATTACGGCTGACTTATTCGTAATATTCATGCAAACGGAGCTTCTCTTTTTTTTATTTGTCTTTATGCCCACATTACCCGA
GGCCTCTACTATGGCTCTTACCTAAACAAAGAAACATGAAATACAGGAGTTGTAATTTTACTTCTTATCATGATAACTGCTTTTGTAGGTTACGTTCTTCCTTGAGGACAA
ATATCTTTTTGGGGGGCAACAGTAATTACTAATCTTCTTTCCGCAATTCCTTTTCTGGGGGAAACTCTTGTACAATGAATTTGAGGCGGGTTTTCAGTAGATAATGCAACT
CTAACCCGATTTTTTGCATTCCATTTCTTACTTCCTTTTGTGGTAGCAGCTATGATCATAGTGCACCTCATTTTCCTCCATGAAACTGGCTCAAACAATCCCGTAGGTCTA
AATTCAAATGCAGATAAGATTTCATTCCACCCTTACTTTATTTATAAAGACTTATTTGGTTTTATGTTATTTTTTTTTCTCCTTACAATATTTGCTCTTTTTTATCCTAAT
CTTCTGGGTGACCCA???
N_whitei
AAACCATCGTTGTTTTCAACTACTAAAACAATAATGGCCCTCCTCCGAAAAACTCACCCTCTTCTTAAACTAGCTAATACCTCTTTAATAGACCTTCCTACACCTCCTAAC
ATCTCATACTGATGAAACTTCGGCTCTCTCTTAGGCATATGCTTAATTGTACAAATCTTAACTGGACTCTTTCTTGCAATACATTACACCCCCGATATTACTATAGCATTT
TCTTCCGTTAACCACATTTGCCGGGATGTCAACCACGGTTGACTCATTCGCAATATTCATGCAAATGGGGCTTCATTTTTCTTTATCTGTATTTATATACATATTGCTCGA
GCTATATACTATGGTTCCTACCTTTACAAAAGCACGTGAAACACGGGAGTTATTTTACTACTCCTTGTAATAGTAACCGCATTCGTTGGTTACGTTCTCCCCTGAGGACAA
ATATCATTCTGA??
??
??
??
N_paraguayensis
??CGAAAAACTTATCCCCTGTTGAAACCAATTAATAATGCCTTAATTGATCTACCCACCCCAATTAAT
ATCTCAACCTGATGAAATTTTGGCTCTCTCCTAGGACTATGCTTAACACTACAAATTTTAACAGGCCTGTTCCTTGCAATACACTATAACTCAGACGTCTCCTCCGCTTTC
TCTTCAGTAGTACATATTTGCCGAGACGTGAACTATGGGTGACTTATTCGAAATATACACGCTAATGGTGCTTCTTTAGCTTTTATCTGCCTCTACCTTCATGTCGGACGG
GGCTTATACTATGGCTCTTATATATATAAAGAAACATGAAGTGTGGGGGTAGTACTCCTCCTTCTTATAATA??????????????????????????????????????
??
??
??
??
A_adloffi
????????????????????????????????ANNNNNNNNNNNNNNNNNNNNNNNNNNNNNNNNNNNNNNNNNNNNNNNNNNNNNNNNNNNNNNNNNNNNNNNNNNNNN

NNNNNNNNNNNNNNNNNNNNNNNNNNNNNNNNNNNNNNNNNNNNNNNNNNNNNNNNNNNNNNNNNNNNNNNNNNNNNNNNNNNNNNNNNNNNNNNNNNNNNNNNNNNNNN
NNNNNNNNNNNNNNNNNNNNNNNNNNNNNNNNNNNNNNNNNNNNNNNNNNNNNNNNNNNNNNNNNNNNNNNNGGGGCTTCTCTTTTTTTTATTTGTCTTTATGCCCATATTACCCGA
GGCCTCTACTATGGCTCTTACCTGTACAAAGAAACATGAAATACAGGGGTTGTAATTTTACTCCTCGTTATGATAACTGCTTTTGTAGGMTACGTTCTCCCTTGAGGGCAG
ATATCTTTTTGGGGAGCAACAGTTATTACTARTCTTCTTTCCGCAATTCCTTTTTTAGGGGAAACTCTTGTACAATGAATTTGAGGGGGGTTTTCAGTAGATAACGCAACT
CTAACCCGATTTTTTGCATTCCATTTCCTATTTCCTTTTGTGGTAGCAGCTATAGTAATGGTACACCTTRTTTTCCTCCATGAAGCTGGCTCAAACAAYCCAGTAGGGCTA
MATTCAAATGCAGATAAGATTTCTTTCCACCCTTRCTTTACCTATAAAGACTTGTTTGGTTTTATATTATTTTTTCTTCTCCTCACAGTATTTGCTCTTTTTTATCCCAAT
CTTCTGGGTGATCCAGAAAATTTTTCCCCAGCCAACCCTYTAATAANNNNNNNNN?????????????????????
A_charrua
???????????????????????????????AATGGCCCC?YTACGAAAAACTCACCCCCTTCTTAAGTTATTTAATAGYGCTTTAGTAGAYCTTCCTACTCCYCCTAAT
ATCTCATTATGATGAAACTTTGGCTCTTTATTAGGCCTTTGYCTAGCAACACAGATCTTAACMGGGCTTTTTTAGCAATACATTATACTCCTGACACCACAATAGCTTTT
TCTTCTGTCGCCCATATTTGTCGCGATGTAAACTACGGGTGACTTATTCGCAATGTTCATGCAAATGGCGCTTCWTTCTTTTTTATTTGTCTTTATGCTCAYATTACTCGA
GGCCTTTACTAYGGCTCCTACCTRTACAAAGAAACATGAAACACCGGGGTCGTTATTTTACTTCTCGTAATGATGACTGCTTTTGTAGGCTACGTTCTTCCTTGAGGGCAG
ATATCTTTTTGGGGGGCAACAGTCATTACGAACCTTCTTTCCGCAATTCCTTTTTTAGGGGAAACTCTTGTACAATGAATTTGAGGGGGTTTCTCAGTAGATAATGCAACT
CTWACCCGATTTTTTGCATTTCATTTCTTATTTCCCTTTGTGGTAATRGCCATAGTTATARTTCACCTAATTTTCCTTCATGAAACTGGCTCTAACAATCCAGTAGGCCTA
AATTCAAATGCAGATAAAATTTCATTCCACCCTTACTTCTTATACAAAGACCTATTTGGTTTCATACTATTTTTTRCCCTTCTCATAATATTTGCTCTTTTTTACCCKAAY
CTTCTAGGTGACCCAGAAAATTTTTCMCCCGCCAACCCCCTAATAANNNNNNNNN?????????????????????
A_wichi
??????????????????????????????????GGCCCCCTTACGTAAAACCCACCCCCTTTTTAAATTATTTAACAATGCTTTAGTAGACCTTCCTACTCCTCCCAAT
ATCTCCTCGTGATGAAATTTGGCTTCATTACTTGGSCTGTGYTTAGCAACACAAGTTTTAACAGGACTTTTTTAGCAATACACTATACCCCTGACACTACAATAGCTTTT
TCATCAGTTATTCACATTTGTCGTGATGTAAATTATGGATGACTCATCCGAAATATTCATGCAAACGGAGCTTCATTCTTTTTTATTKGTCTATATGCTCATGTTTCTCGC
GGGCTTTATTATGGTTCTTATTTATATAAAATAACATGAAACACWGGAGTTGTTATTTTACTCCTTGTAATAATAACTGCCTTTGTAGGGTACGTTCTTCCCTGAGGCCAA
ATGTCTTTTTGGGGAGCAACAGTTATTACTAATCTTCTTTCCGCAATCCCTTTTTTAGGAGAAACTTTAGTGCAATGAATTTGAGGYGGGTTTTCTGTAGATAATGCAACC
CTTACCCGATTCTTCGCATTTCACTTCTTATTACCCTTTATAATTATAGCCCTAATCCTGGTTCACNNNNNNNNNTTACATGAGACTGGCTCCAATAACCCAGTAGGCCTA
AATTCAAATGTAGATAAAATCGCTNNNNNNNNNNNNNNNNNNNNNNNNNNNNNNNNNNNNNNNNNNNNNNNNNNNNNNNNNNNNNNNNNNNNNNNNNNNNNNNNNNNNN
NNNN??
A_viarius
????????????????????????????????NNNNNNNNNNNNNNNNNNNNNNNNNNNNNNNNNNNNNNNNNNNNNNNNNNNNNNNNNNNNNNNNNNNNNNNNNNNNNNNN
NNNNNNNNNNNNNNNNNNNNNNNNNNNNNNNNNNNNNNNNNNNNNNNNNNNNNNNNNNNNNNNNNNNNNNNNNNNNNNNNNNNNNNNNNNNNNNNNNNNNNNNNNNNNNNNNN
NNNNNNNNNNNNNNNNNNNNNNNNNNNNNNNNNNNNNNNNNNNNNNNNNNNNNNNNNNNNNNNNNNNNNNNNNNNNNNNNNNNNNNNNNNNNGCCTTTATGCTCACATCACCCGA
GGCCTTTACTATGGCTCCTACCTGTACAAAGAAACATGAAACACGGGGGTATTAATTTTRCTTCTAGTAATAATAACTGCTTTTGTAGGCTACGTTCTTCCCTGAGGGCAA
ATGTCTTTCTGAGGCGCAACAGTTATTACTAATCTTCTCTCCGCAATTCCTTTTTTAGGGGAAGCTCTTGTACAGTGAATTTGGGGCGGCTTTTCGGTAGACAATGCAACC
CTAACCCGATTTTTTGCATTTCATTTCTTATTTCCTTTTGTAGTAGCAGCTATAGTCATAGTTCACCTAATTTTCCTCCATGAAACTGGTTCAAATAACCCAGCAGGTCTA
AACTCAAACACAGACAAAATTTCATTTCACCCTTATTTTCKGTATAAAGACCTGTTTGGTTTCATGCTAWWTTTTCTTCTCCTCACAATGTTTGCTCTTTTWWACCCYAAT
CTTCTAGGTGACCCAGAAAACTTTTCCCCGGCVAMCYYTNNNNNNNNNNNNNNN?????????????????????
A_bellottii
???????????????????????????????AATGGCCCCT?TACGAAAAACTCACCCYCTTTTTAAACTATTTAACAGCGCTTTRGTWGACCTTCCCACCCCCYCTAAT
ATTTCATCATGATGAAATTTGGCTCTTTACTAGGCCTCTGTCTAGCAACACAGATCTTAACTGGACTTTTTTAGCAATACATTACACCCCSGAYACTGCAATAGCTTTT
TCTTCTGTTGCTCAYATTTGTCGCGATGTAAATTACGGGTGRCTCATCCGTAAYATACATGCAAATGGRGCTTCTCTTTTTTTTATTTGTCTYTATGCCCACATTACCCGG

GGCCTTTACTACGGCTCCTACYTATATAAAGAAACATGAAAYACAGGAGTTRTAATTTTACTCCTAGTAATAATAACTGCTTTTGTAGGTTACGTTCTTCCTTGAGGGCAR
ATGTCCTTTTGAGGAGCAACAGTAATTACTAATCTTCTTTCCGCMATTCCGTTTTTAGGAGAARYTCTTGTTCAATGAATTTGAGGCGGATTTTCAGTAGACAACGCAACT
TTAACYCGWTTCTTTGCATTTCATTTTTTATTCCCCTTTGTAGTAGCAGCTATAGTCATAATTCACCTAATTTTYCTCCATGAAACTGGCTCAAATAATCCAGCAGGCCTA
AATTCAAGCACAGATAAAATTTCCTTYCACCCTTACTTTACTTATAAAGACCTTTTTGGCTTCATACTATTCTTCCTTGTTCTCACAACACTTGCTCTTTTTTACCCTAAT
CTTCTAGGTGAYCCAGANNNNNNNNNNNNNNNNNNNNNNNNNNNNNNNNNNNNNN?????????????????????
A_prognathus
?????????????????????????????????NNNNNNNNNNNNNNNNNNNNNNNNNNNNNNNNNNNNNNNNNNNNNNNNNNNNNNNNNNNNNNNNNNNNNNNNNNNNNNNNNN
NNNNNNNNNNNNNNNNNNNNNNNNNNNNNNNNNNNNNNNNNNNNNNNNACMCAAATCYTRACYGGSCTTTTTTTAGCAATGCACTAYACCCCTGACACYACAATAGCWTTT
TCCTCMGTAGCSCATATTTGTCGCGATGTTAACTACGGGTGACTCATTCGWAATATWCATGCAAACGGAGCTTCTYTYTTYTTTATCTGYCTTTATGCACACATTACCCGA
GGCCTTTACTATGGCTCTTACTTATATAAAGAGACCTGAAATACAGGTGTTGTAATTTTACTCCTAGTAATAATAACWGCTTTYNNNGKSKRCNNNSTWNNNNNNNNNNNN
NNNNNNNNNNNNNNNNNNNNNNNNNNNNNNNNNNNNNNNNNNNNNNNNNNNNNNNNNNNNNNNNNNNNNNNNNNNNNNNNNNNNNNNNNNNNNNNNNNNNNNNNNNNNNNNN
NNNNNNNNNNNNNNNNNNNNNNNNNNNNNNNNNNNNNNNNNNNNNNNNNNNNNNNNNNNNNNNNNNNNNNNNNNNNNNNNNNNNNNNNNNNNNNNNNNNNNNNNNNNNNNNN
NNNNNNNNNNNNNNNNNNNNNNNNNNNNNNNNNNNNNNNNNNNNNNNNNNNNNNNNNNNNNNNNNNNNNNNNNNNNNNNNNNNNNNNNNNNNNNNNNNNNNNNNNNNNNNNN
NNNNNNNNNNNNNNNNNNNNNNNNNNNNNNNNNNNNNNNNNNNNNNNNNNNNNNN?????????????????????
A_affinis
?????????????????????????????????AATGGCCCCCTACGAAAAACCCACCMCSTTTTTAAATTACTAAATAATGCTTTAGTWGAYCTCCCTACCCCYCCCAAT
ATTTCATCATGRTGAAATATGGGCTCTTTATTAGGMCTATGYTTAGCTACTCAARTCTTAACYGGGCTTTTTTTAGCAATACATTATACRCCYGACACCACAATAGCATTC
TCTTCYGTYGCTCACATTTGTCGTGATGTTAATTAYGGTTGACTCATTCGAAAYATTCATGCAAACGGGGCCTCCTTTTTYTTTCTTTGTCTTTATGCTCACATTACTCGA
GGCCTTTACTATGGCTCTTACTTAYAYAAARTAACATGAAAYACTGGTGTAGYWATTTTGCTCCTTGTAATAATRACCGCTTTTGTTGGTTATGTTCTTCCTTGAGGTCAA
ATGTCTTTTTGAGGYNCYACAGTRATTACKAATCTSCTYCCGCTATCCCCTTTYNNNNNNNNNNNNNNNNNNNNNNNNNNNNNNNNNNNNNNNNNNNNNNNNNNNNNNNNN
NNNNNNNNNNNNNNNNNNNNNNNNNTYSMYACTTCCCTTTGTAGTARCAGCTATAGTTCTCGTACACTTAATTTCCTTCATGAAAYGGGCTCCAACAACCCAGCAGGCCTA
AATTCAAAYACAGATAAAATYTCCTTTCATCCCTACTTTTCRTATAAAGACCTTTTTGGTTTTRCACTATTTTTTCTTCTSCTTATATATCTTGKCTTATTTTATCCAAAT
ATTCTAGGAGACCCTGAAAATTTNNNNNNNNNAACCCTYTAATAANNNNNNNNNN?????????????????????
A_gymnoventris
?????????????????????????????????AATGGCCCCCTACGAAATACTCATCCCCTTTTTAAATTACTTAACAGTGCCCTAGTAGATCTTCCCACTCCGCCCAAT
ATCTCATCATGATGAAATTTTSSCTCGTTRSWAGGTCTATGTCTAGYAACCCAAATCWTAAMTGGGCYTTTTTTAGCAATACWCTATACCCCTGACACCGCTWTGGCTTTT
TCTTCTGTGGCTCACATTTGTCGCGATGTAAATTACGGATGGCTCATTCGTAACCTTCATGCAAACGGGGCTTCTCTCTTTTTCATCTGTCTTTATGCTCACGTTACTCGC
GGTCTCTACTACGGCTCTYATTTATATAAAGAAACATGAAACACCGGGGTCGTAATCTTACTACTAGYGATAATAACCGCCTTTGTGGGTTATGTCCTTCCTTGGGGCCAA
ATATCTTTCTGAGGCGCAACAGTTATTACTAATCTTCTCTCCGCGATCCCCTTTTTAGGGGAAGCCCTTGTACAATGAATTTGAGGTGGGTTTTCAGTAGATAACGCAACC
CTAACCCGCTTCTTTGCTTTTCATTTCTTGTTGCCTTTTGTAGTAGCCGCTACTGTTATAATTMATCTAATCTTCCTTCATGAAACTGGCTCAAATAATCCAGCAGGGTTA
AACTCAAGCACAGATAAAATCTCTTTCCACCCTTATTTTACATATAAAGACCTCTTTGGCTTCATACTATTTTTTATATCTCTCACAATATTTGCCCTTTTTTACCCGAAC
CTTATAGGCGACCCAGAAAATTTTGCCCCGGCCAACCCTTTAATAANNNNNNNNNN?????????????????????
A_robustus
?????????????????????????????????NNNNNNNNNNNNNNNNNNNNNNNNNNNNNNNNNNNNNNNNNNNNNNNNNNNNNNNNNNNNNNNNNNNNNNNNNNNNNNNNNN
NNNNNNNNNNNNNNNNNNNNNNNNNNNNNNNNNNNNNNNNNNNNNNNNNNNNNNNNNNNNNNNNNNNNNNNNNNNNNNNNNNNNNNNNNNNNNNNNNNNNNNNNNNNNNNNNN
NNNNCTGTAGCCCACATTTGTCGCGATGTAAATTACGGGTGACTCATTSGTAGTATTCATGCAAATGGGGCTTCTCTTTTTTTCATTTGCCTTTATGCTCATATTACCCGA
GSCCTTTACTACGGCTCTTACTTATATAAAGAAACATGAAATACGGGGGTTATCGTTTTACTTCTGGTGATGATAACTGCTTTKGTAGGCTACGTTCTTCCTTGAGGTCAA
ATATCTTTTTGGGGGGCAACTGTTATCACCAACCTTCTTTCTGCCCTCCCCTTTTTAGGAGAAACCCTTGTTCAATGAATYTGAGGCGGGTTTTCAGTGGATAATGCAACC

CTAACCCGATTCTTTGCATTTCATTTTTTGCTTCCCTTTGTAGTAACAGCTATAGTCATAGTTCACTTAATTTTCCTCCATGAAGCCGGCTCAAATAATCCAGCAGGCCTA
AATTCAAGCACAGACAAANTTTCCTTYNACCCTTANNNNNNNNNNNNNNNNNNNNNNNNNNNNNNNNNNNNNNNNNNNNNNNNNNNNNNNNNNNNNNNNNNNNNNNNNNNNNNN
NNNNNNNNNNNNNNNNNNNNNNNNNNNNNNNNNNNNNNNNNNNNNNNNNNNNNNNN????????????????????
A_elongatus
?????????????????????????????????AATGGCCCCCTASGAAAAACTCATCCCCTTTTTAAATTGCTAAATAGTTCTTTAGTAGACCTTCCCACCCCTCCAAAC
ATTTCATCATGATGAAATTTTGGCTCTTTACTTGGCCTCTGTCTGGCAACACAGATCTTAACCGGGCTTTTTTAGCAATGCACTATACCCCTGACACTTCAATAGCTTTT
TCCTCCGTAGCCCATATTTGTCGCGATGTCAACTACGGATGACTCATTCGTAATATTCATGCAAACGGAGCTTCTCTTTTCTTCATCTGTCTTTATGCACATATTACCCGA
GGCCTTTACTATGGCTCTTACCTGTATAAGGAGACCTGAAATACAGGTGTCATGATCTTACTTTTAGTAATAATAACAGCCTTCGTAGGCTACGTTCTTCCCTGAGGCCAA
ATGTCCTTTTGGGGAGCAACAGTTATTACTAACCTTCTTTCCGCAATTCCTTTTTTAGGCGAAACCCTTGTTCAATGAATCTGAGGCAGCTTTTCAGTAGACAACGCAACC
TTAACCCGGTTTTTCGCATTTCATTTCTTACTGCCCTTTGTGGTAGCAGCTATAGTTATAGTTCACTTAATTTTCCTTCATGAATCCGGCTCTAATAATCCAGCAGAATTN
NNNNNNNNNNNNNNNNNNNNNNNNNNNNNNNNNNNNNNNNNNNNNNNNNNNNNNNNNNNNNNNNNNNNNNNNNNNNNNNNNNNNNNNNNNNNNNNNNNNNNNNNNNNNNNN
NNNNNNNNNNNNNNNNNNNNNNNNNNNNNNNNNNNNNNNNNNNNNNNNNNNNNNNN????????????????????
A_luteoflammulatus
???
ATTTCATCMTGGTGAAACTTTGGCTCTTTACTAGGTCTCTGTCTAGTAACTCAAATCTTAACTGGGCTTTTCTTAGCTATACACTATACCCCTGACACCACAATAGCTTTT
TCTTCTGTCGCCCACATTTGTCGCGATGTAAATTACGGGTGGCTTATTCGTAACCTTCATGCAAATGGGGCTTCTCWCTTTTTCATCTGTCTCTATGCTCATATCACACGA
GGCCTCTACTATGGCTCTTACTTATACAAAGAAACATGAAATACAGGAGTCGTAATTTTACTTCTAGTAATAATAACTGCTTTTGTAGGCTACGTCCTTCCTTGAGGTCAA
ATATCTTTTTGAGGAGCAACAGTTATCACGAACCTTCTTTCCGCAATTCCTTTTTTAGGGGAAGCCCTCGTACAATGAGTCTGAGGGGGGTTTTCAGTGGACAACGCAACC
CTAACCCGATTTTTTTGCATTTCATTTCTTGCTGCCTTTTGTAGTAGCAGCTGTAATCATAATCCACCTAATTTTCCTTCATGAAACTGGCTCAAACAACCCGGTAGGCTTA
AGTTCAAACACAGATAAAATTCCTTTTCACCCTTATTTTATATATAAAGACCTTTTTGGCTTTCTAATATTCTTTCTTCTCCTTACAACCTTCACGCTTTTTTACCCCAAT
CTTTTAGGCGACCCAGAAAACTTTTCTCCTGCAAACCCGTTAATAACACCTCCTC??????????????????????
;

cc + 4 19 21 23 26 29 33 42 64 72;
;
proc
;
