# Supplementary material for: An endangered new species of seasonal killifish of the genus Austrolebias (Cyprinodontiformes: Aplocheiloidei) from the Bermejo river basin in the Western Chacoan Region
Source: PLoS One. 2018 May 16;13(5):e0196261. doi: 10.1371/journal.pone.0196261 (PMC5955519; doi:10.1371/journal.pone.0196261)
Supplement: S3 Appendix — (PDF) [file pone.0196261.s003.pdf]

Table S3. Collecting sites and GenBank accession numbers of Rivulidae sequences used in the present work.

Species	Collection Identification	Collecting site	Mitochondrial *cyt-b* Accession number
Austrolebias adloffi (Ahl, 1922)	GP304	Ponte do Gravataí, Rio Grande do Sul, Brazil	AY724374.1
Austrolebias adloffi	GP308	Ponte do Gravataí, Rio Grande do Sul, Brazil	AY724372.1
Austrolebias affinis (Amato, 1986)	GP470	Route 5, km 399.5, Tacuarembó Department, Uruguay	AF245464.1
Austrolebias alexandri (Castello & Lopez, 1974)	GP475	Salto City, Salto Department, Uruguay	AF245011.1
Austrolebias arachan (Loureiro, Azpelicueta & García, 2004)	GP657	Parque Municipal, Melo City, Cerro Largo Department, Uruguay	AF245466.1
Austrolebias bellottii (Steindachner, 1881)	GP400	Bañado Verocay, Salto Department, Uruguay	AF245007.1
Austrolebias bellottii	R156	Route 11, 21 km S from Río de Oro, Chaco Province, Argentina	FJ826891
Austrolebias charrua (Costa & Cheffe, 2001)	GP350	Route 19, km 7 (pond 33), Rocha Department, Uruguay	AY724390.1
Austrolebias charrua	GP362	Route 19, km 7 (pond 33), Rocha Department, Uruguay	AY724391.1
Austrolebias cheradophilus (Vaz-Ferreira *et al*., 1964)	GP416	Los Sauces, Valizas, Rocha Department, Uruguay	AF245467.1
Austrolebias cinereus (Amato, 1986)	GP2018	F2 from Route 21, Carmelo City, Colonia Department, Uruguay	KF027476
Austrolebias cinereus	GP2541	Route 21, Carmelo City, Colonia Department, Uruguay	KF027477
Austrolebias cinereus	GP735	Route 21, Carmelo City, Colonia Department, Uruguay	AF245005.1
Austrolebias duraznensis (García, Scvortzoff & Hernández, 1995)	GP2396	Next to Mercedes, Soriano Departement, Uruguay	KF192624
Austrolebias duraznensis	GP2397	Next to Mercedes, Soriano Departement, Uruguay	KF192625
Austrolebias duraznensis	GP466	Estadio Rampla, Durazno City, Durazno Department, Uruguay	AF245012.1
Austrolebias elongatus (Steindachner, 1881)	GP2389	Villa Soriano Town, Soriano Department, Uruguay	KF027478
Austrolebias elongatus	GP2545	Villa Soriano Town, Soriano Department, Uruguay	KF027479
Austrolebias elongatus	R159	Azul City, Buenos Aires Province, Argentina	FJ826892
Austrolebias elongatus	R160	Azul City, Buenos Aires Province, Argentina	FJ826893

Species	Collection Identification	Collecting site	Mitochondrial *cyt-b* Accession number
Austrolebias gymnoventris (Amato, 1986)	GP2393	Salamanca Caves, Maldonado Department, Uruguay	KF027480
Austrolebias gymnoventris	GP2394	Salamanca Caves, Maldonado Department, Uruguay	KF027481
Austrolebias gymnoventris	GP490	Route 13, India muerta. Rocha Department, Uruguay	AF245463.1
Austrolebias juanlangi (Costa, Cheffe, Salvia & Litz, 2006)	R003	BR290, Arroio Don Marcos, Rio Grande do Sul, Brazil	AF245460.1
Austrolebias luteoflammulatus (Vaz-Ferreira, Sierra & Paulete, 1964)	GP2391	Route 9, Probides, Rocha Department, Uruguay	KF027482
Austrolebias luteoflammulatus	GP2392	Route 9, Probides, Rocha Department, Uruguay	KF027483
Austrolebias luteoflammulatus	GP2547	Route 9, Probides, Rocha Department, Uruguay	KF027484
Austrolebias luteoflammulatus	GP2549	Route 9, Probides, Rocha Department, Uruguay	KF027485
Austrolebias luzardoi (Perujo, Calviño, Salvia & Prieto, 2005)	R227	Artigas City, Artigas Department, Uruguay	FJ826894
Austrolebias melanoorus (Amato, 1986)	GP894	Route 5, km 399.5, Tacuarembó Department, Uruguay	AY724373
Austrolebias nigripinnis (Regan, 1912)	GP397	Bañado Verocay, Salto Department, Uruguay	AF245013.1
Austrolebias nioni (Berkenkamp, Reichert & Prieto, 1997)	GP677	Route 26, km 331, Tacuarembó Department, Uruguay	AF245457.1
Austrolebias patriciae (Huber, 1995)	R113	Typical locality, Paraguay	FJ826897
Austrolebias periodicus (Costa, 1999)	R261	Route BR 293, next to Don Pedrito,Rio Grande do Sul Estate, Brazil	FJ826898
Austrolebias prognathus (Amato 1986)	GP2378	Route 8, next to Jose Pedro Varela Town, Lavalleja Department, Uruguay	KF027486
Austrolebias prognathus	GP2379	Route 8, next to Jose Pedro Varela Town, Lavalleja Department, Uruguay	KF027487
Austrolebias prognathus	GP381	Route 19, km 6.5, Rocha Department, Uruguay	AF245458
Austrolebias quirogai (Loureiro, Duarte & Zarucki, 2011)	GP2536	Cerro de las cuentas Town, Cerro Largo Department, Uruguay	KF027488
Austrolebias quirogai	GP2537	Cerro de las cuentas Town, Cerro Largo Department, Uruguay	KF027489
Austrolebias quirogai	GP2538	Cerro de las cuentas Town, Cerro Largo Department, Uruguay	KF027490
Austrolebias reicherti (Loureiro & García 2005)	GP1085	Bañados del Este (pond 43), Treinta y Tres Department, Uruguay	AY724398.1
Austrolebias reicherti	GP1104	Bañados del Este (pond 42), Treinta y Tres Department, Uruguay	AY724401.1

Species	Collection Identification	Collecting site	Mitochondrial *cyt-b* Accession number
Austrolebias robustus (Günther, 1883)	R046	Route 63, Km 16.2, Dolores City, Buenos Aires Province, Argentina	FJ826899
Austrolebias robustus	R049	Vivoratá City, Buenos Aires Province, Argentina	FJ826900
Austrolebias robustus	R082	Next to 9 de Julio City, Buenos Aires Province, Argentina	FJ826896
Austrolebias vandenbergi (Huber, 1995)	R116	Typical locality, Paraguay	FJ826901
Austrolebias vazferreirai (Berkenkamp, Etzel, Reichert & Salvia, 1994)	GP675	Melo City, Cerro Largo Department, Uruguay	AF245015
Austrolebias vazferreirai	GP809	Melo City, Cerro Largo Department, Uruguay	
Austrolebias viarius (Vaz-Ferreira, Sierra de Soriano & Scaglia de Paulete 1964)	GP377	Intersection of the Routes 10 and 16, Rocha Department, Uruguay	AY724388.1
Austrolebias viarius	GP880	Route 16, km 2.5 (pond 11), Rocha Department, Uruguay	AY724383.1
Austrolebias viarius	GP881	Route 16, km 2.5 (pond 11), Rocha Department, Uruguay	AY724384.1
Austrolebias wolterstorffi (Ahl, 1924)	GP530	Route 15, km 137.6, Lascano City, Rocha Department, Uruguay	AF245014.1
Austrolebias monstrosus (Huber, 1995)	R197	Salta Province, Argentina	FJ826895
Austrolebias patriciae (Huber, 1995)	R113	Typical locality, Paraguay	FJ826897
Austrolebias wichi n.sp	R192	Salta, Argentina	-----------
Austrolebias cyaneus (Amato, 1987)	R003	Arroio Don Marcos. BR 290, RS. BRASIL	AF245461
Austrolebias minuano Costa & Cheffe, 2001	------		KJ475091
Austrolebias nachtigalli Costa & Cheffe, 2006	------		KJ475098
Austrolebias nigrofasciatus Costa & Cheffe, 2001	------		KJ475087
Nematolebias whitei (Myers, 1942)	------		KF311332
Neofundulus paraguayensis (Eigenmann & Kennedy, 1903)	------		AF002510
