# Supplementary figures and images for: An endangered new species of seasonal killifish of the genus Austrolebias (Cyprinodontiformes: Aplocheiloidei) from the Bermejo river basin in the Western Chacoan Region
Source: PLoS One. 2018 May 16;13(5):e0196261. doi: 10.1371/journal.pone.0196261 (PMC5955519; doi:10.1371/journal.pone.0196261)

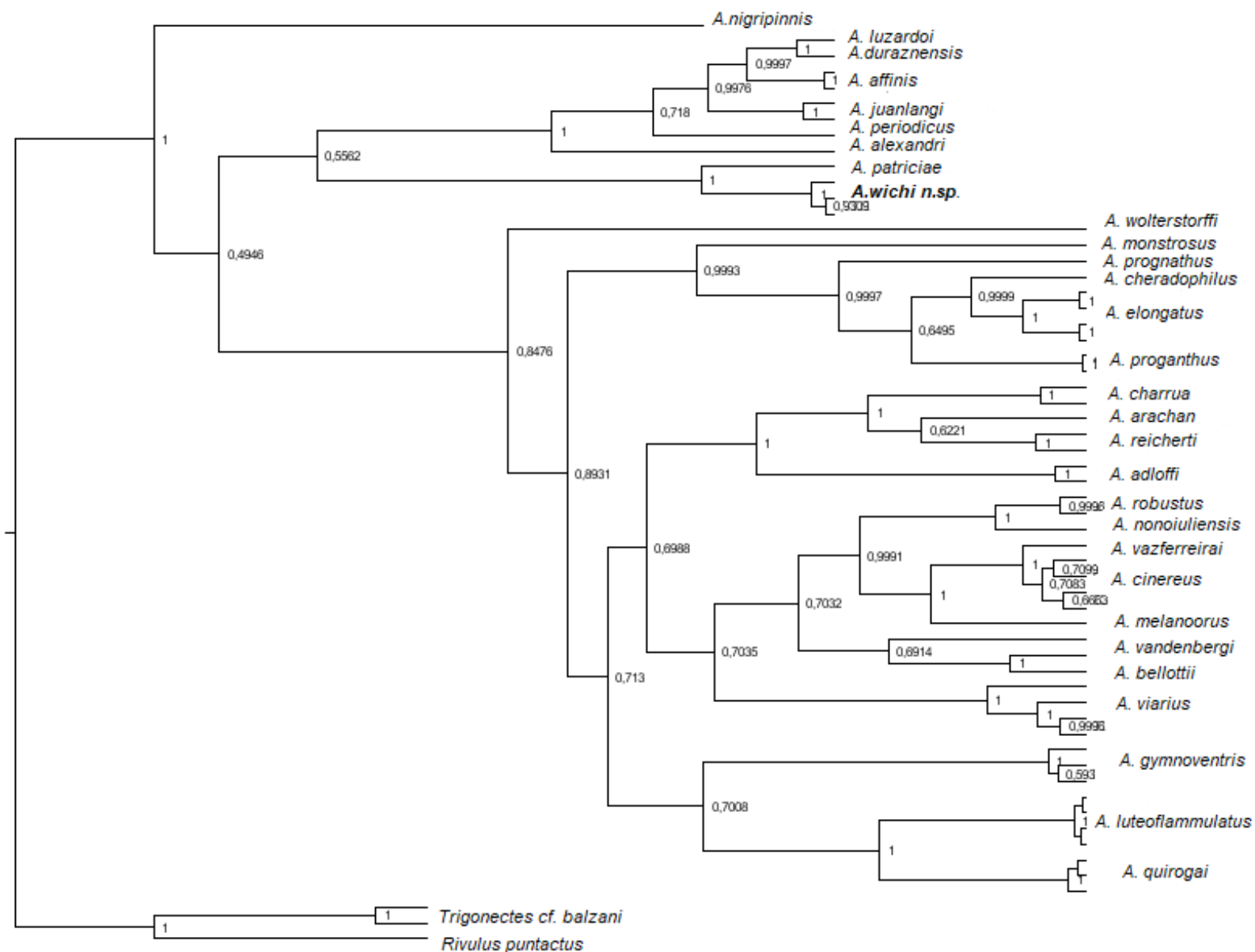

Supplement: S4 Appendix — (PDF) [file pone.0196261.s004.pdf]
